# Reduction of class I histone deacetylases ameliorates ER‐mitochondria cross‐talk in Alzheimer's disease

**DOI:** 10.1111/acel.13895

**Published:** 2023-06-26

**Authors:** Daniela Marinho, Ildete Luísa Ferreira, Ricardo Lorenzoni, Sandra M. Cardoso, Isabel Santana, A. Cristina Rego

**Affiliations:** ^1^ CNC‐Center for Neuroscience and Cell Biology University of Coimbra Coimbra Portugal; ^2^ IIIUC‐Institute for Interdisciplinary Research University of Coimbra Coimbra Portugal; ^3^ CIBB‐Center for Innovative Biomedicine and Biotechnology University of Coimbra Coimbra Portugal; ^4^ FMUC‐Faculty of Medicine University of Coimbra Coimbra Portugal; ^5^ Neurology Department CHUC‐Centro Hospitalar e Universitário de Coimbra Coimbra Portugal

**Keywords:** amyloid beta peptide, calcium, histone deacetylases, mitochondria, mitochondrial‐associated ER membranes, tacedinaline

## Abstract

Several molecular mechanisms have been described in Alzheimer's disease (AD), including repressed gene transcription and mitochondrial and endoplasmic reticulum (ER) dysfunction. In this study, we evaluate the potential efficacy of transcriptional modifications exerted by inhibition or knockdown of class I histone deacetylases (HDACs) in ameliorating ER‐mitochondria cross‐talk in AD models. Data show increased HDAC3 protein levels and decreased acetyl‐H3 in AD human cortex, and increased HDAC2‐3 in MCI peripheral human cells, HT22 mouse hippocampal cells exposed to Aβ_1–42_ oligomers (AβO) and APP/PS1 mouse hippocampus. Tacedinaline (Tac, a selective class I HDAC inhibitor) counteracted the increase in ER‐Ca^2+^ retention and mitochondrial Ca^2+^ accumulation, mitochondrial depolarization and impaired ER‐mitochondria cross‐talk, as observed in 3xTg‐AD mouse hippocampal neurons and AβO‐exposed HT22 cells. We further demonstrated diminished mRNA levels of proteins involved in mitochondrial‐associated ER membranes (MAM) in cells exposed to AβO upon Tac treatment, along with reduction in ER‐mitochondria contacts (MERCS) length. HDAC2 silencing reduced ER‐mitochondria Ca^2+^ transfer and mitochondrial Ca^2+^ retention, while knockdown of HDAC3 decreased ER‐Ca^2+^ accumulation in AβO‐treated cells. APP/PS1 mice treated with Tac (30 mg/kg/day) also showed regulation of mRNA levels of MAM‐related proteins, and reduced Aβ levels. These data demonstrate that Tac normalizes Ca^2+^ signaling between mitochondria and ER, involving the tethering between the two organelles in AD hippocampal neural cells. Tac‐mediated AD amelioration occurs through the regulation of protein expression at MAM, as observed in AD cells and animal models. Data support transcriptional regulation of ER‐mitochondria communication as a promising target for innovative therapeutics in AD.

## Graphical abstract

Inhibition or silencing of class I HDACs counteract ER‐mitochondria Ca^2+^ dyshomeostasis and mitochondrial function by regulating the expression of Ca^2+^‐handling proteins at MAM in AD cell and animal models. HDAC inhibition also decreases ER‐mitochondria contact length and NFAT transcriptional activity that regulates InsP3R expression, potentially controlling the cross‐talk between the two organelles in AD hippocampus.

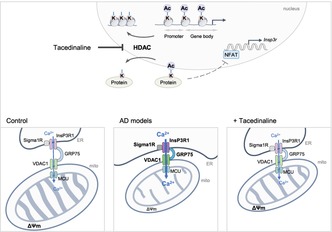

**Abbreviations**
acetyl‐H3: acetylated histone H3
AD: Alzheimer's disease
AβO: amyloid‐beta (Aβ_1–42_) oligomers
Ca^2+^_i_
: intracellular free Ca^2+^
CDR: clinical dementia rating
ECF: enhanced chemifluorescence
ER: endoplasmic reticulum
FCCP: trifluoromethoxy carbonylcyanide phenylhydrazone
GRP75: 75 kDa glucose‐regulated protein
HDACi: histone deacetylase (HDAC) inhibitor
InsP3R: inositol 1,4,5‐trisphosphate receptor
MAM: mitochondrial‐associated ER membranes
MCI: mild cognitive impairment
MCU: mitochondrial Ca^2+^ uniporter
MERCS: mitochondria‐ER contact sites
Mfn: mitofusin
MICOS: mitochondrial contact site and cristae organizing system
MTT: 3‐(4,5‐dimethylthiazol‐2‐yl)‐2,5diphenyltetrazolium bromide
NFAT: nuclear factor of activated T cells
NMDA: *N*‐methyl‐d‐aspartate
NMDAR: NMDA receptor
OMM: outer mitochondrial membrane
PBMCs: peripheral blood mononuclear cells
PDZD8: PDZ domain‐containing protein 8
PI: propidium iodide
PMSF: phenylmethylsulfonyl fluoride
PEG: polyethylene glycol
qRT‐PCR: quantitative real time (RT) PCR
RMD3: regulator of microtubule dynamics protein 3
SAHA: suberoylanilide hydroxamic acid
SB: sodium butyrate
SERCA: sarco/ER Ca^2+^‐ATPase
Sigma1R: sigma‐1 receptor
Tac: tacedinaline
TBS‐T: tris‐buffered saline (TBS) plus Tween‐20
TEM: transmission electron microscopy
TMRM^+^
: tetramethylrhodamine methyl ester
VAPB: vesicle‐associated membrane protein‐associated protein B
VDAC1: voltage dependent anion channel 1
ΔΨm: mitochondrial transmembrane potential

## 1 INTRODUCTION

Alzheimer's disease (AD) is an age‐related neurodegenerative disease and the most common form of dementia, characterized by the deposition of insoluble aggregates of amyloid‐beta peptide (Aβ) and hyperphosphorylated tau, forming senile plaques and neurofibrillary tangles, respectively, particularly in the hippocampus (Knopman et al., 2021). Early diagnosis of AD ranging from mild cognitive impairment (MCI), in which patients have pure memory impairment, to dementia stages progressing to global cognitive decline and loss of autonomy has been defined in recent years (Jack et al., 2018).

Aβ_1–42_ oligomers (AβO), described as the most neurotoxic amyloid form, induce disruption of intracellular Ca^2+^ homeostasis mediated by *N*‐methyl‐d‐aspartate receptors (NMDAR) containing the GluN2B subunit (Ferreira et al., 2012). AβO‐induced immediate activation of NMDAR enhances mitochondrial Ca^2+^ accumulation in a mechanism involving endoplasmic reticulum (ER) Ca^2+^ release through inositol 1,4,5‐trisphosphate receptor (InsP3R) and mitochondrial depolarization (Ferreira et al., 2015), revealing increased susceptibility of glutamatergic synapses and mitochondrial dysfunction in AD.

Mitochondria‐associated ER membranes (MAM) are highly regulated dynamic contacts that sustain crucial neuronal function, namely Ca^2+^ and protein homeostasis, mitochondrial dynamics and ATP production (Resende et al., 2022 for review). Indeed, disturbances in mitochondrial function have been largely associated with upregulated MAM function, augmented cross‐talk between these two organelles (Area‐Gomez et al., 2012; Hedskog et al., 2013) and increased expression of Ca^2+^ channels (InsP3R and voltage dependent anion channel 1 [VDAC1]), which could be explained by abnormal Aβ interaction with mitochondria (Area‐Gomez et al., 2012; Chu et al., 2021; Hedskog et al., 2013). Furthermore, AβO downregulates the expression of the ER Ca^2+^ sensor STIM2 in a compensatory manner, impairing the neuronal store‐operated Ca^2+^ entry pathway, which further contributes to abnormal intracellular Ca^2+^ signaling observed in AD models (Zhang et al., 2015). Moreover, MAM proteins related with ER‐associated protein degradation, oxidative stress response, mitochondrial protein transport and ATP production were shown to be deregulated in the cortex of APP/PS1 mice prior to memory deficits (Völgyi et al., 2018).

Transcriptional deregulation has been described in AD pathogenesis, related to repressed expression of genes regulating mitochondrial biogenesis (Sheng et al., 2012), vesicle trafficking, cell adhesion, actin cytoskeleton dynamics (Sebollela et al., 2012) and antioxidant defense (Caldeira et al., 2013 for review). These observations may be associated with overexpression of histone deacetylases (HDACs), particularly nuclear HDAC2 and HDAC3 (class I) and cytosolic HDAC6 (class II) (Ding et al., 2008; Gräff et al., 2012; Zhu et al., 2017), although decreased HDAC levels were also reported in AD brains (Pascoal et al., 2022). Moreover, nuclear epigenome was shown to communicate with mitochondria in a bidirectional way, in which mitochondria mediates epigenetic processes and conversely, changes in epigenome regulate mitochondrial function (Matilainen et al., 2017).

HDACs inhibitors (HDACis), namely sodium butyrate (SB) and suberoylanilide hydroxamic acid (SAHA), which inhibit both class I and II HDACs, were shown to be effective in reverting Aβ pathology and the underlying cognitive decline (Fernando et al., 2020; Kilgore et al., 2010). Nevertheless, the influence of transcriptional modulation on mitochondrial abnormalities still remains to be elucidated in AD, as most of the studies have been focused on neuroprotection against oxidative stress (Choi et al., 2015; Gaisina et al., 2018; Rivieccio et al., 2009). Moreover, we have previously observed that SB rescued mitochondrial bioenergetic deficits (Naia et al., 2017) and improved mitochondrial‐dependent Ca^2+^ handling (Oliveira et al., 2006) in cell and mouse models of Huntington's disease. Tacedinaline (Tac; CI‐994), a synthetic benzamide‐based HDACi selective to class I HDACs (Beckers et al., 2007), was shown to improve cognitive and memory dysfunction in an intellectual disability mouse model (Cooper et al., 2020) and in C57BL/6 aged mice (McClarty et al., 2021) and exerted neuroprotective effects in mice spinal cord and brain injury (Sada et al., 2020; Zhang et al., 2018). Furthermore, a crucial physiologic function of class I HDACs in modulating mitochondrial homeostasis was described in *Caenorhabditis elegans* (Shao et al., 2020).

Therefore, we hypothesized that reduction of class I HDACs activity plays a protective role in AD pathogenesis by ameliorating transcriptional activity related with mitochondrial function and inter‐organelle communication. Here we report, for the first time, that Tac or class I HDACs knockdown in AD hippocampal neural cell models counteract ER‐mitochondria Ca^2+^ dyshomeostasis by regulating the expression of Ca^2+^‐handling proteins that control the cross‐talk between the two organelles. Tac‐induced regulation of ER/MAM proteins is further observed in the hippocampus of AD animals. These findings support ER‐mitochondrial‐related transcriptional regulation as a promising target for innovative therapeutics in AD.

## 2 RESULTS

### 2.1 Tac and SB, but not SAHA, protect against AβO‐induced cytotoxicity

**FIGURE 1:**
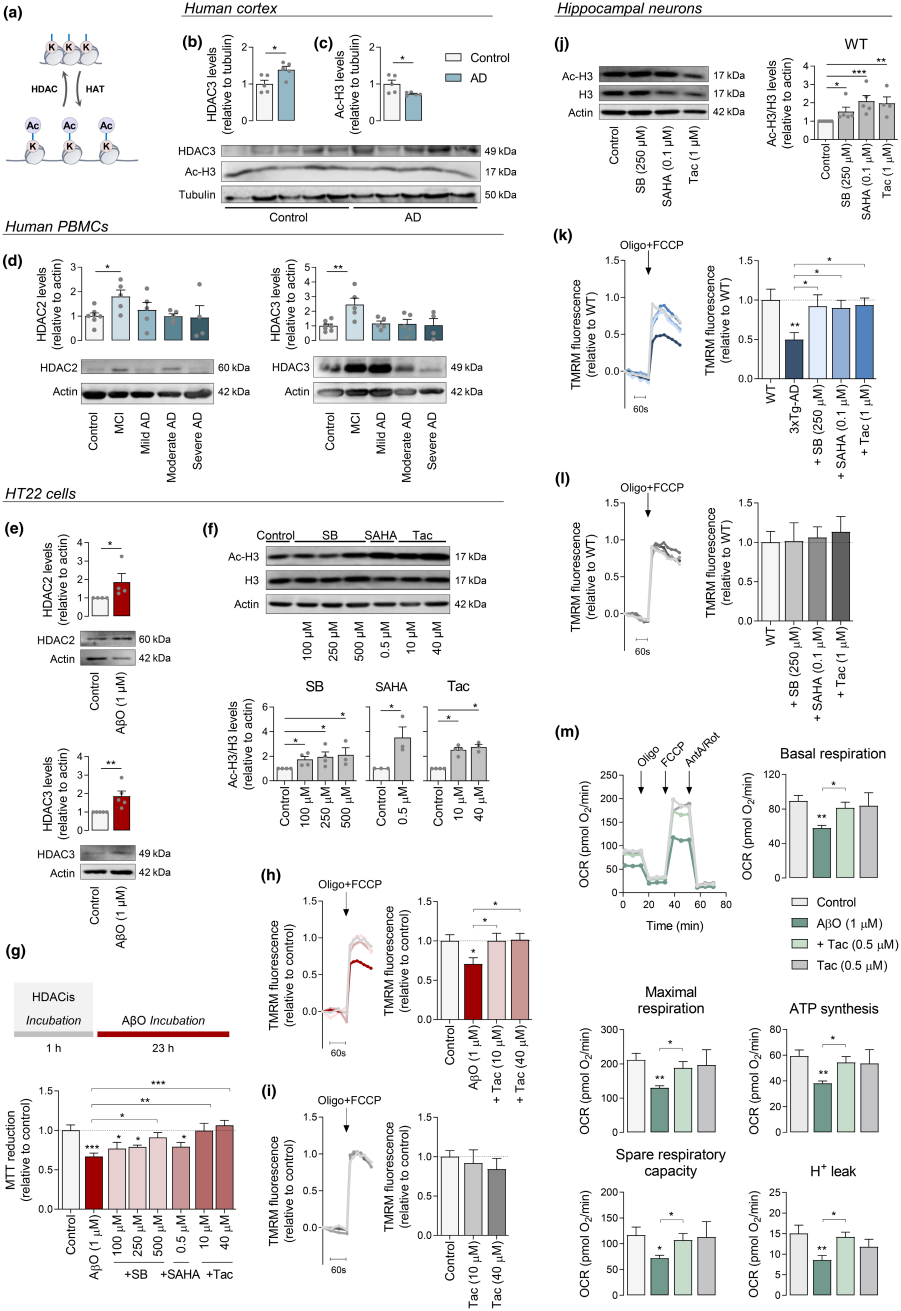
Levels of HDAC2 and HDAC3 and effect of HDACis on acetyl‐H3 levels, AβO‐induced toxicity, and mitochondrial function. Scheme of histone acetylation/deacetylation (a) Western blotting analysis of HDAC3 (b) and acetyl‐H3 (c) protein levels in postmortem brain cortex derived from control individuals or AD patients. Data are the mean ± SEM of 5 individuals *per* group. Western blotting analysis of HDAC2 and HDAC3 protein levels in PBMCS derived from control individuals, MCI or AD patients. Data are the mean ± SEM of 4–10 individuals *per* group (d). HT22 cells were treated for 23 h with 1 μM AβO. Western blotting analysis of HDAC2 and HDAC3 protein levels (e). Cells were treated for 24 h with HDACis (SB, SAHA and Tac at the indicated concentrations). Western blotting analysis of acetyl‐H3 protein levels (f). Cells were pre‐exposed for 1 h with HDACis and then co‐treated with 1 μM AβO for the remaining 23 h of incubation. Cell metabolic activity was measured using the MTT assay (g). Representative TMRM^+^ fluorescence trace and peak amplitude after oligo plus FCCP stimulation in the presence (h) or absence (i) of AβO. Hippocampal neurons were treated for 24 h with HDACis (250 μM SB; 0.1 μM SAHA or 1 μM Tac). Western blotting analysis of acetyl‐H3 protein levels (j). Representative TMRM^+^ fluorescence trace and peak amplitude in response to maximal mitochondrial depolarization induced by oligo plus FCCP (2 μg/mL; 2 μM) in 3xTg‐AD (k) and WT (l) neurons. WT hippocampal neurons were pre‐treated for 1 h with Tac (0.5 μM) and then co‐incubated with 1 μM AβO for the remaining 23 h. Basal respiration, maximal respiration, ATP production (oligomycin‐sensitive respiration), spare respiratory capacity, and H^+^ leak were quantified using a Seahorse analyzer (m). HT22 cells (f–i) were pre‐treated for 1 h with Tac (10 or 40 μM) and then co‐incubated with 1 μM AβO for the remaining 23 h. Representative F340/380 fluorescence trace and peak amplitude after oligo plus FCCP stimulation in the presence (h) or absence (i) of AβO. Data are the mean ± SEM of 3–7 independent experiments, run in triplicates to quadruplicates. Statistical analysis: Kruskal–Wallis followed by uncorrected Dunn's multiple comparison test, one‐way ANOVA followed by uncorrected Fisher's LSD multiple comparison test, Mann–Whitney test and unpaired Student's *t*‐test; *p < 0.05; **p < 0.01; ***p < 0.001 when compared to the control.

Class I HDACs (Figure 1a), particularly HDAC2 and HDAC3, regulate synaptic plasticity and memory formation and are abundantly expressed in AD human and mouse brains (e.g., Gräff et al., 2012; Zhu et al., 2017). Accordingly, we found increased HDAC3 levels in the cortex of AD patients at Braak stages III–IV, as compared to control individuals (Figure 1b), along with a significant reduction in acetylated histone H3 (acetyl‐H3) levels (Figure 1c). Because HDACs are also expressed in PBMCs (Zhang et al., 2011) and these peripheral cells were previously shown by us to be affected in early AD human and mouse stages, correlating with changes in cerebral cortex (Mota et al., 2015), we determined the levels of HDAC2 and HDAC3 in PBMCs derived from MCI subjects (Clinical Dementia Rating (CDR) of 0.5, as described in Mota et al., 2015) or AD patients with increasing degrees of cognitive impairment. Both HDAC2 and HDAC3 protein levels were significantly increased in MCI, as compared with age‐matched control individuals; however, no differences were observed in PBMCs from mild, moderate, or severe AD stages (Figure 1d). Notably, exposure of HT22 cells to AβO for 23 h caused a significant increase in both HDAC2 and HDAC3 proteins levels (Figure 1e), suggesting a potential role of these deacetylases as transcriptional regulators due to their nuclear localization. These results support the hypothesis that class I HDACs (particularly HDAC2 and HDAC3) are possible therapeutic targets in AD, which can be mimicked by AβO exposure.

Therefore, we evaluated the neuroprotective efficacy of class I HDACis in AD hippocampal neural cells. Hippocampal neurons obtained from WT or 3xTg‐AD mice or mouse hippocampal‐derived HT22 cells were exposed to HDACis, SB, SAHA, and Tac for 24 h, and screened for cell metabolic activity, cell proliferation, and apoptotic/necrotic cell death (Figure S1B,E–G). Exposure to HDACis caused a decrease in MTT reduction in a concentration‐dependent manner in WT hippocampal neurons (Figure S1B) and HT22 cells (Figure S1E), while non‐toxic concentrations tested in WT neurons caused no major effects in 3xTg‐AD hippocampal neurons either (Figure S1B). Because HDACis can arrest cell growth (e.g., Marks et al., 2000), we analyzed whether 24 h treatment with SB, SAHA or Tac affects cell proliferation; however, no differences were observed between control and HDACis‐treated cells (Figure S1F). Moreover, treatment with HDACis increased the levels of acetyl‐H3 in both hippocampal neurons (Figure 1j, Figure S1C) and HT22 cells (Figure 1f).

A decrease in MTT reduction was observed in HT22 cells subjected to 1 μM AβO (Figure 1g), indicating a compromised metabolic activity, which was not accompanied by changes in cell death, as evaluated by double Hoechst/PI staining (Figure S1G). AβO‐induced metabolic compromise in HT22 cells was completely abolished in the presence of Tac (at 10 and 40 μM) (Figure 1g). In addition, cell viability remained unchanged when HT22 cells were incubated with Tac alone (Figure S1G). SB at 500 μM was able to revert cytotoxic effects exerted by AβO (Figure 1g), a concentration shown to cause a significant decrease in cell metabolic activity (about 17%; Figure S1E). In contrast, SAHA (0.5 μM) did not protect HT22 cells against AβO‐induced cell dysfunction (Figure 1g).

### 2.2 HDACis decrease mitochondrial Ca^2+^ retention in 3xTg‐AD hippocampal neurons subjected to NMDAR activation and AβO‐treated HT22 cells

Mitochondrial transmembrane potential (ΔΨm), a key indicator of mitochondrial activity required for ATP production, is reported to be reduced in several AD cell models (e.g., Jadiya et al., 2019). Our results evidence decreased ΔΨm in 3xTg‐AD hippocampal neurons (Figure 1k) and in HT22 cells following AβO treatment (Figure 1h), as revealed by the decrease in TMRM^+^ fluorescence following complete mitochondrial depolarization, when compared with WT neurons. HDACis, namely SB (250 μM), SAHA (0.1 μM), and Tac (1 μM), completely reverted ΔΨm dissipation in 3xTg‐AD neurons (Figure 1k). Moreover, Tac (10, 40 μM) prevented mitochondrial depolarization induced by exposure to AβO in HT22 cells (Figure 1h). Evaluation of O_2_ consumption rate (OCR), another readout of mitochondrial function, revealed that the basal and maximal respiration, ATP synthesis, spare respiratory capacity, and H^+^ leak were decreased in hippocampal neurons exposed to AβO and that Tac prevented these effects (Figure 1m). However, no changes in coupling efficiency or non‐mitochondrial respiration were observed in AβO‐treated neurons in the absence or in the presence of Tac, as compared to control (Figure S1D). Importantly, ΔΨm, mitochondrial respiration and ATP synthesis remained unchanged in WT neurons and control cells after inhibition of class I HDAC's activity (Figure 1i,l,m).

**FIGURE 2:**
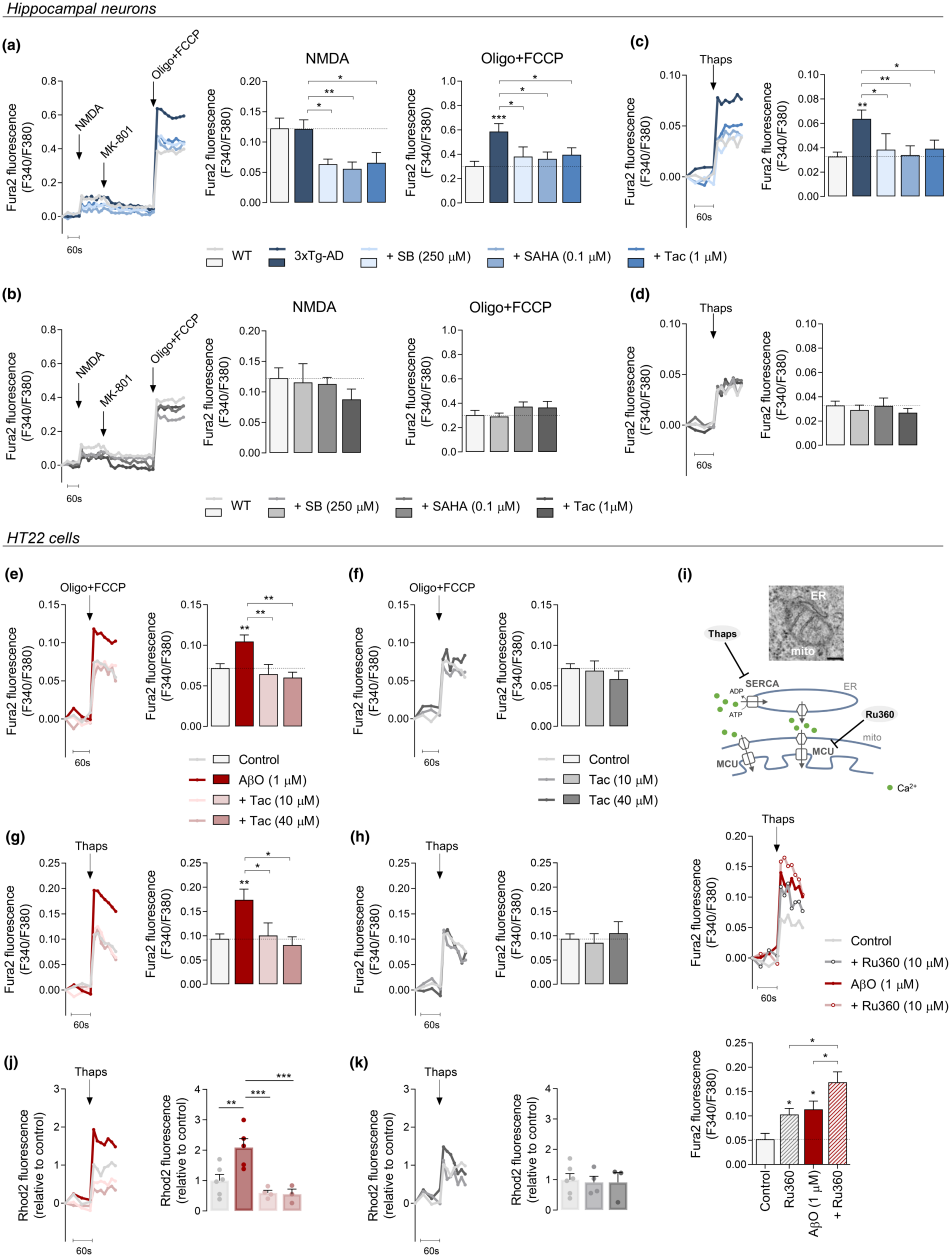
Effect of HDACis on ER‐mitochondria Ca^2+^ transfer in hippocampal neurons and AβO‐treated cells. Hippocampal neurons (a–d) were treated for 24 h with HDACis (250 μM SB; 0.1 μM SAHA or 1 μM Tac). Representative F340/380 fluorescence trace and peak amplitude in response to 100 μM NMDA stimulation and to maximal mitochondrial depolarization induced by oligo plus FCCP (2 μg/mL; 2 μM) in 3xTg‐AD (a) and WT (b) neurons following HDACis pre‐treatment. Representative F340/380 fluorescence trace and peak amplitude in response to ER‐Ca^2+^ storage depletion induced by thapsigargin (1 μM) in 3xTg‐AD (c) and WT (d) neurons. HT22 cells (e–k) were pre‐treated for 1 h with Tac (10 or 40 μM) and then co‐incubated with 1 μM AβO for the remaining 23 h. Representative F340/380 fluorescence trace and peak amplitude after oligo plus FCCP stimulation in the presence (e) or absence (f) of AβO. Representative F340/380 fluorescence trace and peak amplitude following thapsigargin stimulus in the presence (g) or absence (h) of AβO. Representative F340/380 fluorescence trace and peak amplitude in response to thapsigargin stimulation following a pre‐incubation with Ru360 (MCU inhibitor; 10 μM) (i). Representative Rhod2 fluorescence trace and peak amplitude after thapsigargin stimulation in the presence (j) or absence (k) of AβO. Data are the mean ± SEM of 3–8 independent experiments, run in triplicates to quadruplicates. Statistical analysis: Kruskal–Wallis followed by uncorrected Dunn's multiple comparison test, one‐way ANOVA followed by uncorrected Fisher's LSD multiple comparison test; *p < 0.05; **p < 0.01; ***p < 0.001 when compared to control.

Another feature of early neuronal dysfunction in AD is Aβ‐mediated impairment of glutamatergic neurotransmission and subsequent mitochondrial Ca^2+^ dyshomeostasis (Ferreira et al., 2015). Considering this, hippocampal neurons derived from 3xTg‐AD (which produce and secrete Aβ [Vale et al., 2010]) and WT mice were directly subjected to selective activation of NMDAR by stimulation with 100 μM NMDA plus glycine in Mg^2+^‐free pyruvate‐based medium and mitochondrial Ca^2+^ levels measured directly or indirectly using the mitochondrial‐Ca^2+^ sensitive probes Rhod2‐AM (Figure S2A) or Fura2‐AM (Figure 2a), respectively. Our results show increased mitochondrial Ca^2+^ retention in 3xTg‐AD compared with WT neurons (Figure 2a, Figure S2A) in response to Ca^2+^ entry involving GluN2B‐containing NMDAR (Figure S2B). Interestingly, we observed no significant differences in Ca^2+^_i_ levels after NMDA stimulation in 3xTg‐AD compared to WT neurons (Figure 2a), suggesting that increased mitochondrial Ca^2+^ retention occurs independently of changes in Ca^2+^ influx through the NMDAR. We further ascertained if mitochondrial Ca^2+^ dyshomeostasis could occur independently of NMDAR activation. We found that under basal conditions (in the absence of NMDAR activation), mitochondrial‐Ca^2+^ levels in 3xTg‐AD neurons were similar to WT neurons (Figure S2C). These observations point to a mechanism whereby NMDAR activation leads to increased Ca^2+^ retention in mitochondria in 3xTg‐AD hippocampal neurons.

Thus, we examined the effect of HDACis on intracellular Ca^2+^ homeostasis in hippocampal neurons subjected to NMDAR activation. Ca^2+^_i_ levels evoked by NMDA plus glycine were shown to be decreased following SB, SAHA, and Tac incubation in 3xTg‐AD neurons (Figure 2a); in contrast, no differences in NMDAR‐evoked Ca^2+^ entry were found in HDACis‐treated WT neurons (Figure 2b). These data suggest an inhibitory effect of HDACis at NMDAR level in 3xTg‐AD neurons. Importantly, the increase in mitochondrial Ca^2+^ levels upon NMDA stimulation observed in 3xTg‐AD neurons was significantly counteracted by HDACis pre‐exposure (Figure 2a), whereas no effect was observed in WT neurons (Figure 2b). Taken together, these results suggest that HDACis regulate NMDA‐induced Ca^2+^_i_ levels as a compensatory mechanism to prevent mitochondrial Ca^2+^ overload promoted by NMDAR activation in 3xTg‐AD hippocampal neurons. In contrast, basal mitochondrial Ca^2+^ levels remained unaltered following incubation with SB, SAHA, or Tac in both WT and 3xTg‐AD neurons (Figure S2C,D).

To better understand the effect of class I HDACs inhibition on Aβ‐mediated mitochondrial Ca^2+^ dyshomeostasis, mitochondrial Ca^2+^ measurements were also performed in HT22 cells exposed to AβO. Data show that AβO significantly enhanced mitochondrial Ca^2+^ retention, when compared to control cells, which was completely reverted upon treatment with Tac (Figure 2e). Conversely, SB or SAHA had no effect on mitochondrial Ca^2+^ levels in AβO‐treated HT22 cells (Figure S2E). In addition, we found no differences in control cells after Tac (Figure 2f), SB or SAHA treatment (Figure S2F).

### 2.3 Tac prevents enhanced mitochondrial Ca^2+^ accumulation from ER in AβO‐treated HT22 cells

Because ER is one of the largest Ca^2+^ stores in cells, ER‐Ca^2+^ levels were evaluated by Fura2 fluorescence in hippocampal neurons and HT22 cells subjected to thapsigargin, a selective non‐competitive inhibitor of sarco/ER Ca^2+^‐ATPase (SERCA) that disrupts Ca^2+^ homeostasis in this cellular compartment. ER‐Ca^2+^ levels were significantly increased in 3xTg‐AD hippocampal neurons (Figure 2c), as compared to WT neurons, as well as in HT22 cells upon AβO treatment (Figure 2g). Although Tac and the pan‐HDACis (SB and SAHA) treatment counteracted the enhanced ER‐Ca^2+^ accumulation in 3xTg‐AD hippocampal neurons (Figure 2c), no differences were found on HT22 ER‐Ca^2+^ levels in the presence of AβO plus pan‐HDACis (Figure S2G). In contrast, Tac, which effect is described to be selective to class I HDACs, decreased Ca^2+^ retention into ER in AβO‐treated cells (Figure 2g), again suggesting a specific regulation of AβO‐induced abnormalities in intracellular Ca^2+^ buffering. Moreover, no effect on ER‐Ca^2+^ levels was observed in WT neurons (Figure 2d) nor in HT22 control cells (Figure 2h, Figure S2H) following HDACis treatment.

Taking into account that ER interacts with mitochondria at MAM to control intracellular Ca^2+^ signaling, we assessed Ca^2+^ communication between the two organelles with Ru360 (10 μM), a selective inhibitor of mitochondrial Ca^2+^ uniporter (MCU) and thus of mitochondrial Ca^2+^ uptake, in cells subjected to ER‐Ca^2+^ release, in the presence of thapsigargin. Remarkably, Ru360 increased cytosolic Ca^2+^ levels after ER‐Ca^2+^ release as compared with control cells (Figure 2i), indicating Ca^2+^ transfer into mitochondria. Furthermore, Ca^2+^_i_ rise was significantly higher in AβO‐treated cells (Figure 2i), indicating enhanced mitochondrial Ca^2+^ uptake from ER in cells subjected to AβO exposure. These findings suggest that Aβ promotes mitochondrial Ca^2+^ overload associated with mitochondrial dysfunction via enhanced ER‐Ca^2+^ release, potentially triggered by excessive Ca^2+^ stored in the ER.

To determine the influence of HDACis in precluding intracellular Ca^2+^ dyshomeostasis, we measured mitochondrial Ca^2+^ levels with Rhod2‐AM in response to thapsigargin stimulation in HT22 cells. Our results show that AβO exposure elicited an increase in Ca^2+^ retained in mitochondria following ER‐Ca^2+^ release, which was significantly prevented in HT22 cells treated with Tac (Figure 2j). No alterations in mitochondrial Ca^2+^ were observed in control cells subjected to Tac (Figure 2k), indicating that HDACis (specifically Tac) control ER‐mitochondria cross‐talk and function.

### 2.4 Tac ameliorates the expression of MAM‐related proteins and MAM morphology in AβO‐treated HT22 cells

**FIGURE 3:**
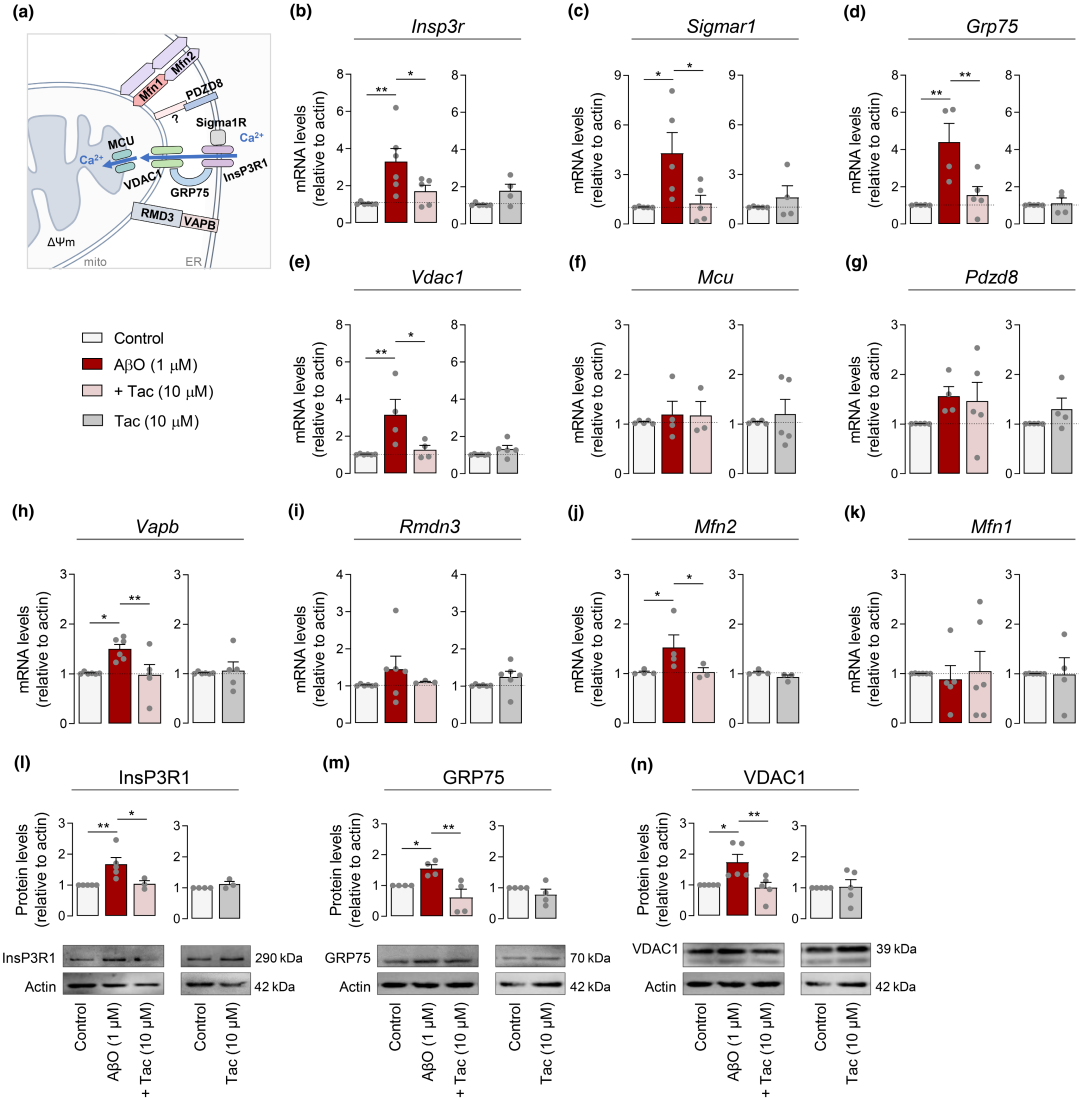
Tac effect on MAM‐related proteins in AβO‐treated cells. Schematic representation of proteins at MAM (a). HT22 cells were pre‐treated for 1 h with Tac (10 μM) and then co‐incubated with 1 μM AβO for the remaining 23 h or treated solely with Tac for 24 h. Relative expression of *Insp3r* (b), *Sigmar1* (c), *Grp75* (d), *Vdac1* (e), *Mcu* (f), *Pdzd8* (g), *Vapb* (h), *Rmdn3* (i), *Mfn2* (j), and *Mfn1* (k) mRNA. Western blotting analysis of InsP3R1 (l), GRP75 (m), and VDAC1 (n) protein levels. Actin was used as the housekeeping messenger and the loading control. Data are the mean ± SEM of 3–6 independent experiments, run in triplicates to quadruplicates. Statistical analysis: Kruskal–Wallis test followed by uncorrected Dunn's multiple comparisons test, one‐way ANOVA followed by uncorrected Fisher's LSD multiple comparison test, Mann–Whitney test and unpaired Student's *t*‐test; *p < 0.05; **p < 0.01 when compared to control.

In order to dissect transcriptional modifications exerted by Tac on MAM‐related proteins in AβO‐treated HT22 cells, mRNA and related protein levels were evaluated by qRT‐PCR and Western blotting analysis, respectively. InsP3R, a major ER Ca^2+^‐release channel, which is stabilized by sigma‐1 receptor (Sigma1R), enables Ca^2+^ transfer into mitochondria through a complex with 75 kDa glucose‐regulated protein (GRP75) and mitochondrial VDAC1 (Figure 3a). Considering that disturbances in mitochondrial function have been mainly associated with upregulated MAM function and augmented cross‐talk between ER and mitochondria (Area‐Gomez et al., 2012; Hedskog et al., 2013), we evaluated whether the alterations in Ca^2+^ signaling observed in AβO‐treated cells correlated with changes in the expression of Ca^2+^‐handling proteins present at MAM, including InsP3R1, Sigma1R, GRP75, VDAC1, PDZ domain‐containing protein 8 (PDZD8), and MCU. Our results evidence that exposure to AβO led to increased mRNA levels of *Insp3r* (Figure 3b), *Sigmar1* (Figure 3c), *Grp75* (Figure 3d), and *Vdac1* (Figure 3e). However, we did not observe changes in mRNA levels of mitochondrial Ca^2+^ uniporter (*Mcu*) (Figure 3f), known to be largely responsible for mitochondrial Ca^2+^‐uptake in AD neurons (e.g., Ferreira et al., 2015), nor in the ER‐mitochondria tethering protein *Pdzd8* (Figure 3g), which regulates cytoplasmic Ca^2+^ dynamics and consequently Ca^2+^ uptake by mitochondria (Hirabayashi et al., 2017), in response to AβO treatment. Interestingly, we found upregulated expression of other MERCS components with a physiological structural function, like vesicle‐associated membrane protein‐associated protein B (*Vapb*) (Figure 3h) and mitofusin 2 (*Mfn2*) (Figure 3j), in AβO‐treated cells, while regulator of microtubule dynamics protein 3 (*Rmdn3*, also known as protein tyrosine phosphatase interacting protein 51, PTPIP51) (Figure 3i) and *Mfn1* (Figure 3k), that interact with VAPB and Mfn2, respectively, to form MAM‐tethering complexes, showed similar mRNA levels to those found in control cells. Concordantly with mRNA levels, InsP3R1, GRP75, and VDAC1 protein levels were found to be increased in HT22 cells treated with AβO (Figure 3l–n). Upregulated expression of ER‐mitochondria Ca^2+^‐transfer proteins was completely prevented in AβO‐treated cells following Tac treatment (Figure 3b–n), but was not affected in control cells (Figure 3b–n).

**FIGURE 4:**
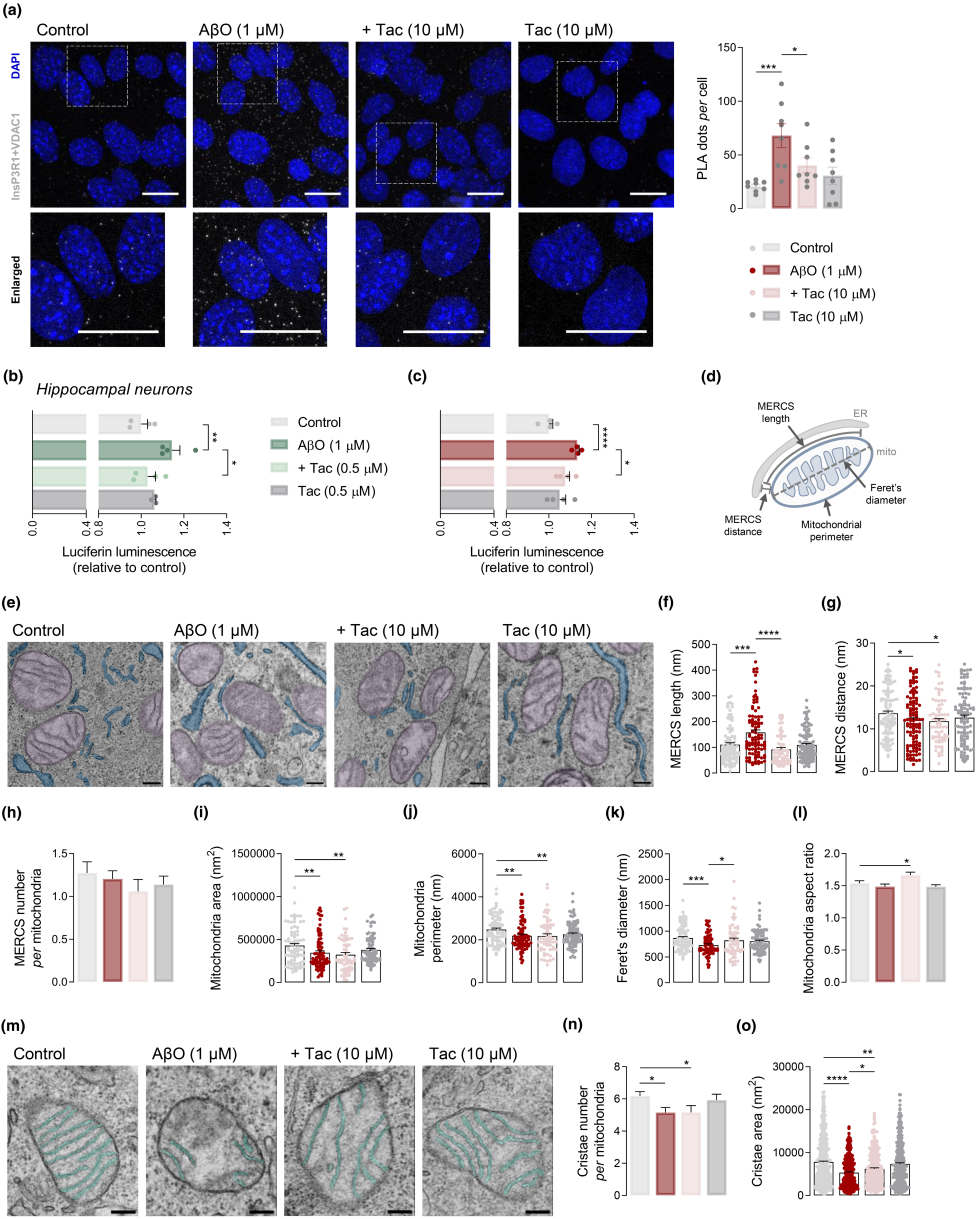
Tac effect on InsP3R1‐VDAC1 interaction and MERCS morphology in AβO‐treated cells. HT22 cells were pre‐treated for 1 h with Tac (10 μM) and then co‐incubated with 1 μM AβO for the remaining 23 h or treated solely with Tac for 24 h. Representative confocal images of in situ PLA signal (gray) indicating a physical interaction between InsP3R1 and VDAC1 (scale bar = 20 μm). The lower panels represent enlargements of the boxed areas in the upper panels. Quantification of number of PLA puncta in about 200–240 cells from 8 image stacks per condition (a). WT hippocampal neurons were pre‐treated for 1 h with Tac (0.5 μM) and then co‐incubated with 1 μM AβO for the remaining 23 h or treated solely with Tac for 24 h. NFAT transcriptional activity evaluated by luciferase reporter assay in hippocampal neurons (b) and HT22 cells (c). Data are the mean ± SEM of 4 independent experiments, run in triplicates to quadruplicates. Schematic representation of MERCS structural parameters: MERCS length and distance, mitochondrial perimeter and Feret's diameter (d). HT22 cells were pre‐treated for 1 h with Tac (10 μM) and then co‐incubated with 1 μM AβO for the remaining 23 h. Representative TEM images of mitochondria (in purple) in close contact with ER (in blue) (scale bar = 200 nm) in which distances ≤25 nm were considered as contacts (e). MERCS length (f) and distance (g), number of MERCS per mitochondria (h), mitochondrial area (i) and perimeter (j), Feret's diameter (k) and mitochondrial aspect ratio (l). Representative TEM images of mitochondrial cristae (in green) (scale bar = 200 nm) (m). Number of cristae per mitochondria (n), and cristae area (o). About 70–90 individual mitochondria from 10 randomly selected cells obtained from approximately 30 images were assessed in 26–38 independent TEM images. Statistical analysis: Kruskal–Wallis test followed by uncorrected Dunn's multiple comparisons test, one‐way ANOVA followed by uncorrected Fisher's LSD multiple comparison test; *p < 0.05; **p < 0.01; ***p < 0.0001; ****p < 0.0001 when compared to control.

To test the hypothesis that close contacts between InsP3R1 and VDAC1 underlie augmented ER‐mitochondria Ca^2+^ transfer elicited by AβO, we examined the InsP3R1‐VDAC1 interaction by PLA. Our results showed that AβO triggered InsP3R1‐VDAC1 direct interactions, which were prevented by Tac (Figure 4a). However, Tac did not affect the formation of InsP3R1‐VDAC1 complexes in control cells (Figure 4a). These data provide the first evidence for a role of selective class I HDACis (namely Tac) in interfering with the transcription of genes relevant for MAM connectivity and concomitant ER‐mitochondria Ca^2+^ signaling in AD.

Thus, the question arises as to how the ER‐mitochondria Ca^2+^‐transfer complexes are mechanistically regulated by Tac‐induced acetylation of histone (as shown in Figure 1f,j) and of non‐histone proteins (data not shown). Recent reports have shown that deacetylation‐activated Vav1 stimulates nuclear factor of activated T cells (NFAT) pathway (Rodríguez‐Fdez et al., 2020), which mediates InsP3R upregulation in an AD animal model (Shao et al., 2022). Therefore, to explore whether Tac modulates InsP3R1 expression by regulating NFAT, we measured NFAT transcriptional activity using a luciferase reporter plasmid in our AD cell models. We found a slight but significant increase in NFAT transcriptional activity in AβO‐treated hippocampal neurons (Figure 4b) and HT22 cells (Figure 4c), which was abolished by Tac (Figure 4b,c). No changes in NFAT activity were observed in Tac‐treated neurons (Figure 4b) or HT22 cells (Figure 4c).

To further characterize the effect caused by class I HDACs inhibition on MAM morphology, we performed ultrastructural analysis of HT22 cells using TEM. MERCS were defined by the ER‐mitochondria proximity within 25 nm and the MERCS length was quantified as the length of ER segments within 25 nm distance from the outer mitochondrial membrane (OMM) (Figure 4d). Electron micrographs analysis in AβO‐treated cells showed increased length of the ER‐mitochondria interface (Figure 4e,f) and a narrower gap between the OMM and ER membrane (Figure 4e,g), when compared to control cells. In contrast, the number of contacts between the two organelles, in relation to the number of mitochondria, remained unaltered (Figure 4e,h), indicating that the close apposition between ER and mitochondria relies on expression of intracellular Ca^2+^ signaling‐regulating proteins. Importantly, treatment with Tac in cells exposed to AβO induced a redistribution of ER in close contact with mitochondria by decreasing the MERCS length (Figure 4e,f), without affecting the distance between the organelles (Figure 4e,g) or the MERCS number per mitochondria (Figure 4e,h). ER‐mitochondria interactions in Tac‐treated cells, not exposed to AβO, were similar to control cells (Figure 4e–h). These results suggest that class I HDACis decrease the frequency of tighter interactions between ER and mitochondria, while ameliorates Ca^2+^ transfer into the mitochondrial matrix in AβO‐treated cells.

Additionally, we assessed if Tac treatment could also modulate mitochondrial morphology, specifically mitochondrial size (perimeter and surface area), shape (aspect ratio, defined as the ratio between major and minor mitochondrial axis), fragmentation (Feret's diameter, defined as longest distance between any two points in the mitochondria; Gillmore et al., 2022; Figure 4d), and cristae organization (number and surface area). Mitochondria from AβO‐treated cells exhibited decreased mitochondrial area (Figure 4e,i) and perimeter (Figure 4e,j), as well as reduced Feret's diameter (Figure 4e,k), indicating mitochondrial fragmentation. In contrast with the morphologic evidence of enhanced fission, no significant changes were observed in mitochondrial aspect ratio (Figure 4e,l). Additionally, mitochondria were shown to have a reduced number of cristae (Figure 4m,n) with reduced surface area (Figure 4m,o) in the presence of AβO. Interestingly, we found that while mitochondria were smaller as evaluated by area and perimeter values following Tac treatment in the presence of AβO (Figure 4e,i,j), when compared to control cells, Tac was able to prevent mitochondrial fragmentation (Figure 4e,k), which was accompanied by a significant increase in aspect ratio (Figure 4e,l). Furthermore, Tac treatment caused mitochondrial cristae remodeling by increasing the surface area (Figure 4m,o) without affecting the number of cristae *per* mitochondria (Figure 4m,n). None of the mitochondrial morphological features were altered following treatment with Tac alone (Figure 4e,i–o).

### 2.5 HDAC2‐siRNA prevents enhanced ER‐mitochondria Ca^2+^ transfer while HDAC3‐siRNA decreases ER‐Ca^2^ ^+^ retention in AβO‐treated HT22 cells

**FIGURE 5:**
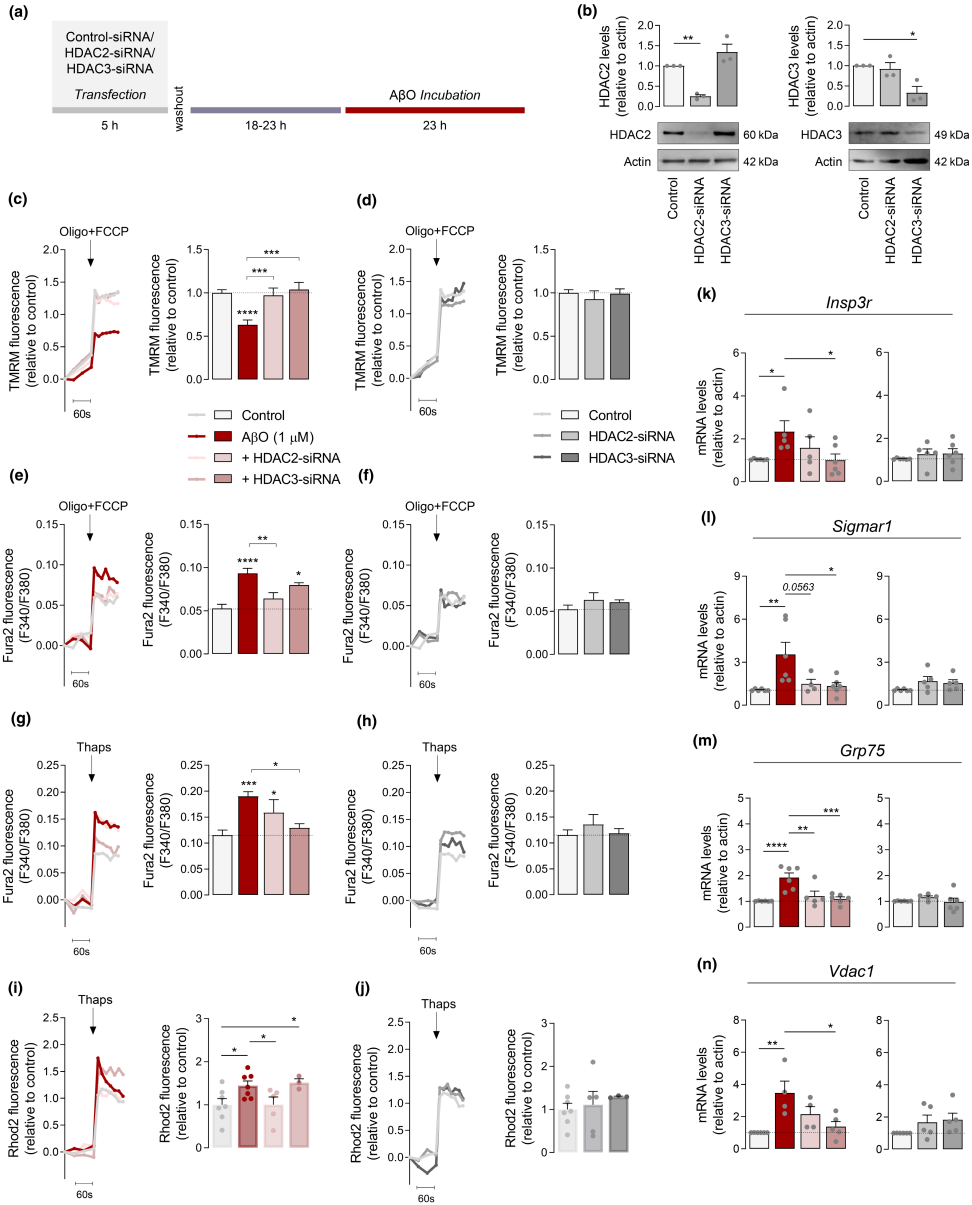
HDAC2/3 knockdown effect on mitochondrial membrane potential, Ca^2+^_i_ levels and MAM proteins in AβO‐treated cells. HT22 cells were transfected with either HDAC2‐ or HDAC3‐siRNA. Scrambled siRNA was used as a control. After transfection, cells were treated with 1 μM AβO and incubated for additional 24 h (a). Western blotting analysis of HDAC2 and HDAC3 proteins levels 48 h after transfection with HDAC2‐ or HDAC3‐siRNA (b). Representative trace of TMRM^+^ fluorescence and peak amplitude relative to control in response to maximal mitochondrial depolarization induced by oligo (2 μg/mL) plus FCCP (2 μM) in the presence (c) or absence (d) of AβO. Representative F340/380 fluorescence trace and peak amplitude after oligo plus FCCP stimulus in the presence (e) or absence (f) of AβO. Representative F340/380 fluorescence trace and peak amplitude in response to ER‐Ca^2+^ storage depletion induced by thapsigargin in the presence (g), or absence (h) of AβO. Representative Rhod2 fluorescence trace and peak amplitude after thapsigargin stimulation in the presence (i) or absence (j) of AβO. Relative expression of *Insp3r* (k), *Sigmar1* (l), *Grp75* (m), and *Vdac1* (n) mRNA. Actin was used as the housekeeping messenger and the loading control. Data are the mean ± SEM of 3–7 independent experiments, run in triplicates to quadruplicates. Statistical analysis: Kruskal–Wallis test followed by uncorrected Dunn's multiple comparisons test and one‐way ANOVA followed by uncorrected Fisher's LSD multiple comparison test; *p < 0.05; **p < 0.01; ***p < 0.001; ****p < 0.0001 when compared to control.

Neuroprotective effects observed following Tac treatment may arise from the combined inhibition of HDAC1, 2, 3, and 8 (class I HDACs). As both nuclear HDAC2 and HDAC3 are upregulated in the hippocampus of AD mouse models and human patients (Gräff et al., 2012; Zhu et al., 2017), we further investigated the impact of these HDACs on mitochondrial function and ER‐mitochondria cross‐talk. Thus, HDAC2 or HDAC3 expression were downregulated by using specific HDAC‐siRNAs in HT22 cells subjected to AβO (Figure 5a). Nontarget siRNA was used as control. In these conditions, quantitative analysis showed a 75% selective reduction in HDAC2 levels and a 67% decrease in HDAC3 levels in cells transfected with HDAC2‐ or HDAC3‐siRNA, respectively (Figure 5b).

Of relevance, AβO‐mediated mitochondrial depolarization was completely precluded after HDAC2 and HDAC3 silencing (Figure 5c), but not in control cells (Figure 5d). Further mitochondrial functional assays showed that HDAC2‐siRNA substantially attenuated the increased Ca^2+^ levels accumulated in mitochondria in AβO‐treated cells (Figure 5e). However, HDAC3 knockdown did not significantly alter mitochondria Ca^2+^ retention induced by AβO (Figure 5e). These observations suggest that in the presence of AβO, HDAC3‐siRNA is able to prevent mitochondrial dysfunction independently of mitochondrial Ca^2+^ overload. Next, we determined whether selective silencing of nuclear HDACs altered ER‐Ca^2+^ accumulation. HDAC3‐siRNA abolished the augmented ER‐Ca^2+^ retention induced by AβO, whereas HDAC2‐siRNA did not cause alterations in the ER‐Ca^2+^ levels in AβO‐treated cells (Figure 5g). In agreement with reduced mitochondrial Ca^2+^ accumulation, Ca^2+^ transfer from ER to mitochondria was markedly decreased in HDAC2 knockdown cells treated with AβO, but not in cells exposed to HDAC3‐siRNA (Figure 5i). Together, these results suggest a differential role of nuclear HDACs in regulating ER and mitochondria Ca^2+^ signaling under AβO exposure. No significant differences were found in ER or mitochondrial Ca^2+^ levels following selective HDAC knockdown in cells non‐exposed to AβO (Figure 5f,h,j).

In addition, we assessed the influence of HDAC2 and HDAC3 silencing on mRNA levels of *Insp3r*, *Sigmar1*, *Grp75*, and *Vdac1*. HDAC3‐siRNA led to reduced expression of *Insp3r* (Figure 5k), *Sigmar1* (Figure 5l), *Grp75* (Figure 5m), and *Vdac1* (Figure 5n) in AβO‐treated cells. Selective HDAC2 knockdown in the presence of AβO significantly decreased mRNA levels of *Grp75* (Figure 5m) and showed a tendency to reduce *Sigmar1* mRNA levels (Figure 5l), but did not induce significant changes in the expression of *Insp3r* (Figure 5k) or *Vdac1* (Figure 5n). These data suggest that HDAC2 and HDAC3 act in a cooperative manner in regulating mitochondrial function and ER‐mitochondria cross‐talk.

### 2.6 Tac decreases Aβ levels and alters the expression of MAM‐related proteins in the hippocampus of APP/PS1 mice

**FIGURE 6:**
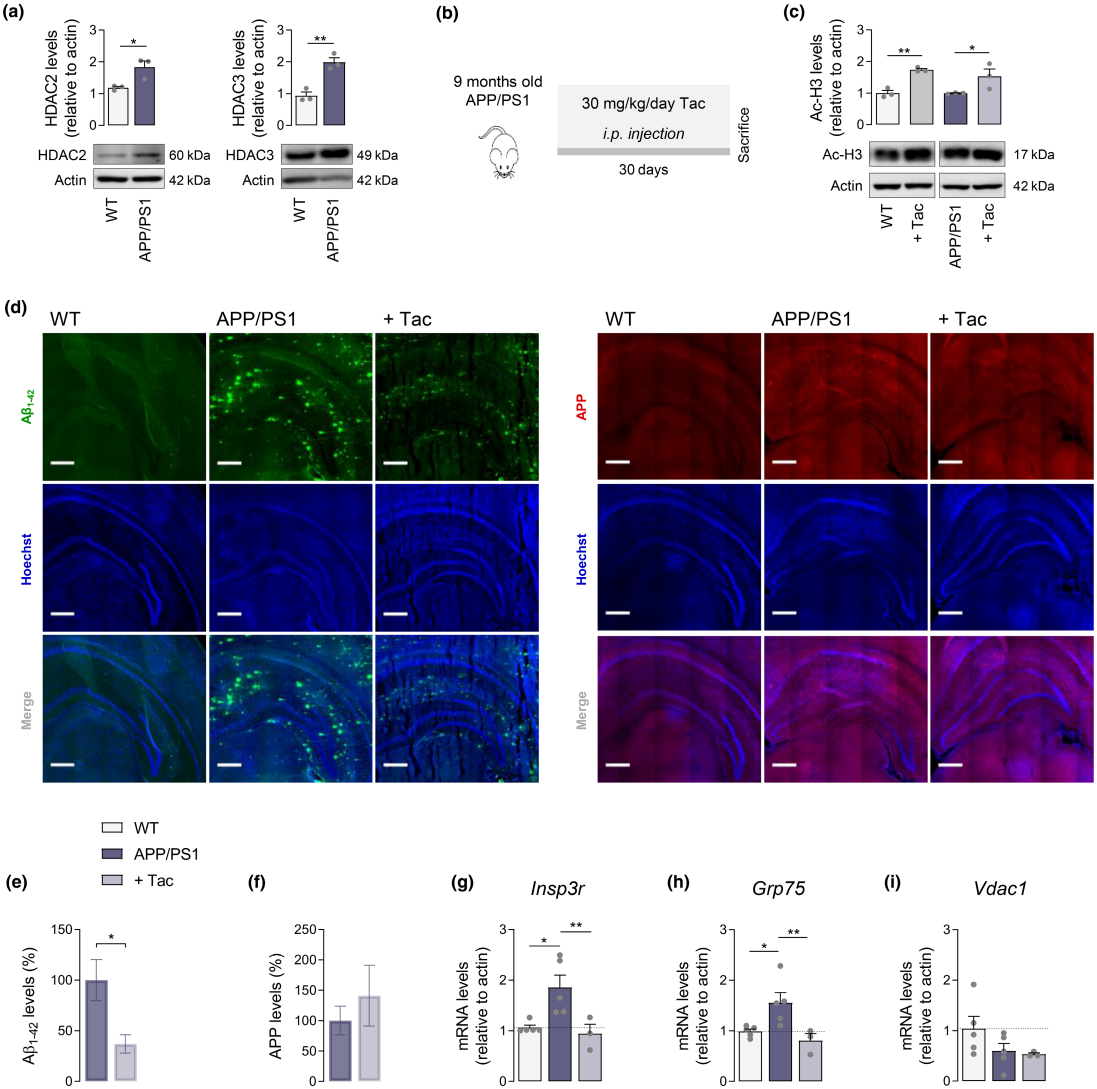
Effect of Tac on Aβ levels and expression of genes involved in ER‐mitochondria Ca^2+^ communication in the hippocampus of APP/PS1 mice. Western blotting analysis of HDAC2 and HDAC3 protein levels in 10 months old APP/PS1 and WT mice (a). APP/PS1 versus WT mice were treated with Tac (30 mg/kg/day) for 30 days (b). Western blotting analysis of acetyl‐H3 protein levels (c). Representative immunofluorescent images (d; scale bar = 100 μm; 25–30 coronal sections) and quantification of Aβ (e) and APP (f) levels. Relative expression of *Insp3r* (g), *Grp75* (h), and *Vdac1* (i) mRNA. Actin was used as the housekeeping messenger and the loading control. Data are the mean ± SEM of 3–5 mice *per* group. Statistical analysis: Kruskal–Wallis test followed by uncorrected Dunn's multiple comparisons test, one‐way ANOVA followed by uncorrected Fisher's LSD multiple comparison test and unpaired Student's *t*‐test; *p < 0.05; **p < 0.01 when compared to control.

Our in vitro results indicate that both pharmacologically inhibiting class I HDACs with Tac and silencing both HDAC2 and HDAC3 constitute relevant neuroprotective strategies in AD hippocampal neural cells. Hence, we sought to determine if the alterations in ER‐mitochondrial cross‐talk induced by Tac could be recapitulated in vivo by using the APP/PS1 mouse model. We initially observed an increase in the levels of nuclear HDAC2 and HDAC3 in the hippocampus of 10 month old APP/PS1 mice, compared to WT mice (Figure 6a), similarly as observed in human AD cortex and MCI PBMCs and HT22 cells exposed to AβO (Figure 1). To examine the neuroprotective effect of class I HDACis in vivo, APP/PS1 mice versus WT aged 9 month‐old were treated with 30 mg/kg/day Tac by intraperitoneal injection for 30 days (until 10 months of age) (Figure 6b). Mice body weight was maintained throughout the experiment (data not shown). Both genotypes exhibited significantly increased acetyl‐H3 levels in the hippocampus upon Tac treatment in comparison with mice treated with vehicle (Figure 6c).

An ELISA‐based assay indicated increased levels (~17‐fold) of soluble Aβ_1–42_ in APP/PS1 mice aged 6–13 month‐old as compared to WT (data not shown). We further assessed the effect of Tac treatment on Aβ levels in APP/PS1 mouse hippocampus by immunofluorescence. A significant reduction in Aβ was observed following Tac treatment, when compared with vehicle‐treated APP/PS1 (Figure 6d,e), suggesting alterations in either Aβ formation or clearance. No Aβ accumulation was found in WT mice (Figure 6d,e). Furthermore, no significant differences in APP levels were observed in the hippocampus of Tac‐treated APP/PS1, compared to APP/PS1 mice, while the presence of APP was scarce in WT mice (Figure 6d,f). Tac‐mediated unaltered APP levels preclude the hypothesis of potential changes in APP expression.

Additionally, we analyzed the expression of genes involved in Ca^2+^ signaling at MAM. Increased mRNA levels of *Insp3r* (Figure 6g) and *Grp75* (Figure 6h) were detected in the hippocampus of APP/PS1 as compared to WT mice, which was completely reverted by Tac (Figure 6g,h). In addition, no significant differences in the expression of *Vdac1* were observed (Figure 6i). These results suggest that inhibition of class I HDACs abolishes early transcriptional dysregulation of key protein players involved in ER‐mitochondria Ca^2+^ cross‐talk, concomitantly with reducing Aβ pathology, in APP/PS1 transgenic mice.

## 3 DISCUSSION

This work is the first to support evidence for epigenetic regulation exerted by selective nuclear class I HDACs, namely HDAC2 and HDAC3, as a relevant neuroprotective strategy for ER and mitochondrial dysfunction and deregulated Ca^2+^ handling in AD pathology. HDAC2 and HDAC3 have been mostly implicated in regulating neuroplasticity and cognitive function. As such, they were shown to be enhanced in AD mouse models, as well as in postmortem brain samples from sporadic AD patients (Gräff et al., 2012; Zhu et al., 2017), as shown in our study. Conversely, a significant reduction of class I HDACs (HDACs 1, 2, and 3) was recently reported in brains from MCI and AD patients, as evaluated by PET analysis using [^11^C]Martinostat tracer, as well as in transgenic rats with both amyloid and tau pathology, but not in aged transgenic rats (McGill‐R‐Thy1‐APP rats with the APP Swedish and Indiana mutations) expressing single amyloid pathology (Pascoal et al., 2022). In addition, HDAC1 activity was shown to be reduced in the hippocampus of aged 5XFAD transgenic mice (Pao et al., 2020), a mouse model characterized by the presence of thioflavin‐positive tau aggregates apart from amyloid plaque accumulation (Shin et al., 2021). These findings suggest HDACs reduction as a possible epigenetic AD signature downstream tau pathology, along disease progression. Of relevance, our results demonstrate that PBMCs from MCI individuals exhibited elevated HDAC2 and HDAC3 levels, but no alterations were observed in AD patients already in the stage of dementia, suggesting that peripheral epigenetic dysregulation associated with histone acetylation imbalance occurs in early pre‐dementia phase of AD (MCI) where patients develop a pure memory deficit while preserving full autonomy. Moreover, we found increased HDAC3 levels, concomitantly with diminished H3 acetylation, in postmortem cortex of AD patients at Braak stages III–IV, which was associated with CDR of 0.5 and very mild dementia (Therriault et al., 2022). Consistent with this study, *Drosophila larvae*, modeling early human AD neurodegeneration, exhibited enhanced HDAC2 in the brain before Aβ deposition (Beaver et al., 2022). Importantly, we also observed increased HDAC2 and HDAC3 protein levels following AβO treatment in HT22 cells and in APP/PS1 transgenic mice, supporting the importance of targeting class I HDACis in ameliorating cognitive function and preventing neuronal dysfunction. SB treatment, which acts through inhibition of both class I and II HDACs, was shown to improve memory in APP/PS1 mice (Govindarajan et al., 2013; Kilgore et al., 2010), to reduce amyloid burden in 5xFAD mouse model (Fernando et al., 2020), and to restore learning behavior and enhance neurogenesis in PS1/2 cDKO mice, an Aβ‐independent mouse model of AD (Cao et al., 2018). Tac, a novel benzamide‐based HDACi that completely inhibits class I HDACs and to a smaller extent HDAC6 (Beckers et al., 2007), was shown to improve cognition in an intellectual disability syndrome mouse model (Cooper et al., 2020), to increase neuronal plasticity after traumatic brain injury (Sada et al., 2020), to promote functional recovery following spinal cord injury (Zhang et al., 2018) and to reduce antipsychotic haloperidol‐induced motor side effects and memory impairment in aged mice (McClarty et al., 2021). However, the role of transcription modulation elicited by Tac has not been addressed in neurodegenerative disorders like AD yet.

Neuronal dysfunction in AD has been described to be mediated by AβO‐induced intracellular Ca^2+^ dyshomeostasis through mGluR5 overactivation, leading to elevated ER‐Ca^2+^ accumulation (Zhang et al., 2015), and through GluN2B‐composed NMDAR (Ferreira et al., 2012, 2015), which leads to mitochondrial Ca^2+^ overload as a consequence of ER‐Ca^2+^ release through InsP3R (Ferreira et al., 2015). Accordingly, our data evidence elevated ER‐Ca^2+^ levels and Ca^2+^ accumulation in mitochondria elicited by NMDAR activation in 3xTg‐AD hippocampal neurons, exhibiting increased levels of endogenous Aβ (Vale et al., 2010), as compared to WT neurons. However, unaltered NMDAR‐mediated Ca^2+^ entry and mitochondrial Ca^2+^ levels in non‐stimulated cells were observed, suggesting that increased Ca^2+^ retention into mitochondria, under NMDA stimulation, occurs after Ca^2+^ release from ER independently of Ca^2+^ influx through NMDAR. Interestingly, it was previously shown that Aβ production can occur at MAM, facilitating its regulatory role on ER‐mitochondria communication (Schreiner et al., 2015). We found that HDACis (SB, SAHA, and Tac) reverted excessive ER‐Ca^2+^ levels and subsequent mitochondrial Ca^2+^ retention following NMDAR activation in 3xTg‐AD neurons, which was preceded by a reduction in NMDA‐evoked Ca^2+^ entry, suggesting a compensatory mechanism to prevent intracellular Ca^2+^ dyshomeostasis.

Previously, it was reported that HT22 cells exposed to extracellular Aβ underwent a deleterious effect on mitochondrial morphology and function similar to that observed using mitochondria‐targeted Aβ (Cha et al., 2012). Here, we describe that AβO promotes excessive ER‐Ca^2+^ retention, favoring ER‐mitochondria Ca^2+^ transfer in HT22 cells, pointing out mitochondrial dysfunction as a consequence of the impaired ER function. However, this effect was counteracted by Tac, but not by SB and SAHA, suggesting that inhibition of selective class I HDACs, rather than that exerted by pan‐HDACis (as SB and SAHA), regulates Ca^2+^ signaling between ER and mitochondria promoted by AβO in the absence of an excitotoxic stimulus.

The impairment of ER‐mitochondria Ca^2+^ signaling observed in AβO‐treated cells might be explained by upregulation of mRNA levels of proteins involved in Ca^2+^ signaling at MAM, namely InsP3R, Sigma1R, GRP75, and VDAC1, InsP3R1‐VDAC1 direct interaction and associated augmented ER‐mitochondria coupling, in accordance with previous studies performed in AD cell and animal models before amyloid plaque formation and postmortem AD brains (Chu et al., 2021; Hedskog et al., 2013). Likewise, we found upregulated mRNA levels of MAM‐associated proteins in the hippocampus of presymptomatic APP/PS1 mice exhibiting increased Aβ levels. Interestingly, D'Eletto et al. (2018) demonstrated increased InsP3R‐GRP75 interaction associated with a reduction in MERCS number and ER‐mitochondria Ca^2+^ transfer and augmented distance between organelles, indicating that MAM structure and ER‐mitochondria cross‐talk have a complex non‐linear regulation. In fact, disturbances in ER–mitochondria cross‐talk in AD have been mainly associated with an increased interaction between both organelles (Area‐Gomez et al., 2012), for example, through binding of mutant PS2 with Mfn2, which promotes mitochondrial Ca^2+^ overload, ΔΨm collapse, and apoptosis (Filadi et al., 2016). In line with increased MERCS length upon AβO treatment observed in the present study, other findings showed that ER undergoes significant extension, promoting the formation of additional contacts with mitochondria and Ca^2+^ transfer between the two organelles under pathological conditions caused by DNA damage (Zheng et al., 2018). Interestingly, Bartok et al. (2019) evidenced that tight interactions between ER and mitochondria occur independently of Ca^2+^ flux through InsP3Rs. Notably, we observed that, through regulation of histone acetylation imbalance, Tac ameliorated the expression of MAM proteins involved in ER‐mitochondria Ca^2+^‐transfer, thereby reducing InsP3R1‐VDAC1 interaction, MAM connectivity, and excessive mitochondrial Ca^2+^ uptake in AβO‐treated cells. Recent data evidence that ablation of InsP3Rs in HEK293 cell line abrogated ER‐mitochondrial Ca^2+^ feedback and cell death (Booth et al., 2021). In addition, InsP3R1 silencing rescued exaggerated Ca^2+^ signaling in cortical and hippocampal neurons, and aberrant hippocampal long‐term potentiation and memory deficits in FAD mouse models (Shilling et al., 2014). Moreover, knockdown of GRP75 impaired ER–mitochondrial coupling, provided protection against mitochondrial dysfunction and cell death, and attenuated mitochondrial Ca^2+^ overload in HT22 cells submitted to glutamate‐induced oxidative stress (Honrath et al., 2017). Overall, these observations validate the neuroprotective role exerted by Tac on ER‐mitochondria cross‐talk through modulation of tethering and Ca^2+^ signaling between the two organelles. However, loss of ER‐mitochondria tethering has been described in temporal cortex from AD patients at the final stages of the disease (Lau et al., 2020), and the enhancement of ER‐mitochondria apposition as a neuroprotective mechanism in a *Drosophila* AD model (Garrido‐Maraver et al., 2020). Contrarily, we observed decreased expression of proteins involved in Ca^2+^ signaling at MAM in APP/PS1 hippocampus upon Tac treatment, concomitantly with decreased Aβ deposition.

Given the well‐established mechanism of HDACs inhibition in gene transcription, we surprisingly found that Tac negatively regulates the expression of MAM‐related proteins such as InsP3R1‐VDAC1 complexes in our AD cell and animal models. These changes might be due to Tac‐induced downregulation of NFAT transcriptional activity in AβO‐treated cells, as observed by us, as activated NFAT was shown to be required for InsP3R upregulation in a *Drosophila* AD model (Shao et al., 2022). This is in line with a study reporting that Ca^2+^‐dependent stimulation of NFAT is downmodulated by acetylated VAV1 and promoted by Lys deacetylation of VAV1 (Rodríguez‐Fdez et al., 2020), suggesting Lys acetylation of non‐histone proteins as a target effect of class I HDACis in modulating transcription of ER‐mitochondria cross‐talk in AD pathogenesis. Alternatively, striking findings of a non‐canonical mechanism linking transcription repression to histone hyperacetylation have been reported. These authors demonstrated that HDACis inhibit transcription by redistributing elongation factors to newly acetylated sites, as gene bodies, resulting in reduced occupancy at promoters and enhancers as well as enhancer activity (Cheng et al., 2018; Greer et al., 2015; Marié et al., 2018; Pinz et al., 2015). Collectively, these findings suggest that HDACis‐induced transcription regulation of MAM function may not be limited to a single pathway but rather a broad effect of both histone and non‐histone acetylation, which requires further investigation.

A previous study reported that hippocampal neurons from McGill‐R‐Thy1‐APP transgenic rats exhibited ER swelling and fragmented mitochondrial cristae under conditions of significant Aβ secretion (Martino Adami et al., 2019). Here, we found that extracellular added AβO disrupted mitochondrial morphology through a reduction in mitochondrial surface area, along with increased fission and cristae fragmentation. Our data also indicate that Tac partially restores mitochondrial morphology by increasing mitochondrial elongation and cristae area, maintaining mitochondrial size and number of cristae. The formation of mitochondrial contact site and cristae organizing system (MICOS) in proximity to MERCS was recently found in yeast (Tirrell et al., 2020), whereas Miro‐1 and ‐2, two proteins required for normal mitochondrial cristae architecture, were shown to regulate MERCS structure and function, namely ER‐mitochondrial Ca^2+^ signaling (Modi et al., 2019). These observations provide new insights into the mechanism that regulate ER‐mitochondria cross‐talk, suggesting that transcriptional modulation of MAM function exerted by Tac in cells exposed to AβO may directly contribute to improved mitochondrial cristae organization.

To further investigate the mechanisms underlying the epigenetic modulation exerted by Tac on ER‐mitochondria dysfunction elicited by AβO, we focused on the effect of nuclear HDAC2 and HDAC3. Our results indicate that HDAC2 silencing abrogated the excessive ER‐mitochondrial Ca^2+^ transfer and decreased GRP75 expression in response to AβO exposure. In contrast, HDAC3 knockdown downregulated the expression of Ca^2+^‐handling associated proteins at MAM, namely InsP3R1, Sigma1R, GRP75, and VDAC1, and reduced the elevated ER‐Ca^2+^ levels in AβO‐treated cells. Interestingly, selective knockdown of HDAC2 or HDAC3 exhibited a similar effect on mitochondrial function by preventing mitochondrial depolarization induced by AβO. More recently, HDAC2 shRNA was reported to enhance mitochondrial respiration and elongation, and reduce the levels of secreted Aβ_1–42_ and Aβ_1–40_ in induced pluripotent stem cell‐derived neurons obtained from AD patients fibroblasts (Frankowski et al., 2021).

In conclusion, we show increased levels of HDAC2 and HDAC3 in PBMCs from MCI individuals, AD human brain cortex (Braak staging III–IV) as well as in the hippocampus of APP/PS1 mice and AβO‐treated mouse hippocampal cell line. Data highlight that pharmacologically inhibiting the activity of class I HDACs (including HDAC2 and HDAC3) with Tac and complementary effects of silencing HDAC2 or HDAC3 alleviates mitochondrial dysfunction and the impairment in ER‐mitochondria cross‐talk in AD hippocampal neural cells. Tac modified expression of MAM‐related proteins involved in ER‐mitochondria Ca^2+^‐transfer, affecting MAM connectivity and ameliorating mitochondrial Ca^2+^ handling, as well as partially restoring mitochondrial morphology. These findings reveal MAM transcriptional regulation as a promising target for innovative therapeutics in AD early stages. In this respect, targeting class I HDACs is expected to impact on epigenetic modulation, potentially having a protective role in AD pathogenesis.

## 4 MATERIALS AND METHODS

### 4.1 Materials

Neurobasal medium, B27 supplement, gentamicin, trypsin, and penicillin–streptomycin were purchased from Gibco, Life Technologies. The synthetic Aβ_1–42_ peptide was from Bachem. Tacedinaline (CI994) was purchased from Sigma‐Aldrich or from MedChemExpress. Fluorescent probes Fura2‐AM, Rhod2‐AM, tetramethylrhodamine methyl ester (TMRM^+^), Hoechst 33342 and propidium iodide (PI), and Pluronic acid F‐127 were obtained from Molecular Probes, Invitrogen. Antibodies against acetyl‐H3 (#06‐599), β‐actin (A5316) and β‐tubulin (T7815) were obtained from Sigma‐Aldrich, as well as RPMI‐1640 Medium (R4130), minimum essential medium (MEM M0268), Dulbecco's modified Eagle's medium (DMEM D5648), DMEM D5030, SB, glutamate, glutamine, 3‐(4,5dimethylthiazol‐2‐yl)‐2,5diphenyltetrazolium bromide (MTT), NMDA, ifenprodil, MK‐801, oligomycin, trifluoromethoxy carbonylcyanide phenylhydrazone (FCCP), ionomycin, antimycin, rotenone, phenylmethylsulfonyl fluoride (PMSF), chymostatin, pepstatin A, leupeptin, antipain, paraformaldehyde, and Duolink in situ Red Starter Kit (DUO92101). SAHA was from Biovision. Primary antibodies anti‐GRP75 (#2816), anti‐H3 (#9715), anti‐HDAC2 (#2540), and anti‐HDAC3 (#3949) were from Cell Signaling; anti‐InsP3R1 (19962‐1‐AP) from Proteintech; anti‐VDAC1 (ab14734) and anti‐APP (ab32136) and Coomassie Blue G‐250 were from Abcam and anti‐Aβ (NBP2‐13075) from Novus Biologicals. Thapsigargin was purchased from Tocris Bioscience. Anti‐mouse (#31320) and anti‐rabbit (#31340) IgG secondary antibodies were from Invitrogen. Enhanced chemifluorescence (ECF) substrate and Ficoll‐Paque were obtained from GE Healthcare. Dithiothreitol, NZYol reagent and NZY first‐strand cDNA synthesis kit were from NZYTech. Bradford protein assay reagent and iQ SYBR Green supermix were purchased from BioRad. Pierce BCA protein assay kit was from Thermo Scientific. Control‐, HDAC2‐ and HDAC3‐siRNA were from Santa Cruz Biotechnology. NFAT luciferase reporter was obtained from Addgene. Polyethylene glycol (PEG) 400 was from LabChem Inc. OCT compound embedding medium was from Bio Optica. All other reagents were from analytical grade.

### 4.2 Aβ_1–42_ oligomers preparation

Aβ_1–42_ preparation containing a high percentage of Aβ oligomers and monomers (about 60% and 40%, respectively; Figure S1A) was obtained from synthetic Aβ_1–42_ as previously described (Klein, 2002). In brief, lyophilized Aβ peptide was dissolved to 1 mM in 1,1,1,3,3,3‐hexafluoro‐2‐propanol (HFIP) in pyrogen/endotoxin‐free tubes and incubated at room temperature for 1 h. HFIP was then evaporated overnight in a chemical hood and completely removed under speed vacuum. Dried monomeric peptide film was further resuspended in anhydrous DMSO to a 5 mM final concentration. The peptide was diluted into Ham's F12 medium to a final concentration of 100 μM and incubated at 4°C for 24 h. The preparation was then centrifuged at 14,000 *g* for 10 min at 4°C to remove insoluble aggregates, and the supernatant (corresponding to the oligomeric peptide) collected. Peptide concentration was determined using the BioRad protein dye assay reagent. To confirm oligomer formation and the presence of different Aβ peptide forms, the preparation was evaluated by 6%–15% Tris–Tricine SDS‐PAGE gel electrophoresis and further staining with Coomassie Blue G‐250.

### 4.3 Animals

3xTg‐AD mice on a C57BL6/129S background harboring APPswe, PS1/M146V and tauP301L transgenes (genetically engineered as previously described by Oddo et al., 2003) were gifted from Dr. Frank LaFerla, University of California, Irvine. APP/PS1 mice harboring APPswe and PS1dE9 transgenes were acquired from the Jackson Laboratory (MMRRC stock #34829) and fully backcrossed onto a B6C3F1/J background. Offspring were genotyped by PCR amplification of genomic DNA extracted from ear clippings. Mice were bred and maintained under a constant temperature, humidity and 12 h light/dark cycle at CNC‐Faculty of Medicine animal house. All procedures were performed in order to minimize exposure to stress and animal suffering, in accordance with EU Directive 2010/63/UE. The study was approved by the Food and Veterinary Directorate under reference 003927_0421/000/000/2018.

### 4.4 Cell cultures

#### 4.4.1 Hippocampal neuronal culture

Primary cultures of hippocampal neurons were prepared from 17 days embryos derived from 3xTg‐AD or WT strain mice. The hippocampi were dissected and collected into pre‐cooled Ca^2+^, Mg^2+^‐free Hank's balanced salt solution (HBSS containing: 137 mM NaCl, 5.36 mM KCl, 0.44 mM KH_2_PO_4_, 4.16 mM NaHCO_3_, 0.34 mM NaH_2_PO_4_.H_2_O, 5 mM glucose, 5.36 mM Hepes, 1 mM pyruvate, pH 7.2) and then digested in 0.003% (w/v) trypsin for 3 min at 37°C. HBSS containing 5% heat‐inactivated FBS was used to halt enzymatic digestion. Following two washing steps with HBSS, the cells were dissociated mechanically in plating medium (MEM supplemented with 10% horse serum, 0.6% (w/v) glucose and 1 mM pyruvate) and plated onto poly‐d‐lysine‐coated plates, at a final density of 5 × 10^4^ cells/cm^2^. After 2 h, the plating medium was replaced by Neurobasal medium supplemented with 2% (v/v) B27, 0.5 mM glutamine, 50 μg/mL gentamicin and 25 μM glutamate. Neuronal cultures were maintained at 37°C in a humidified incubator chamber with 95% air and 5% CO_2_ for 10–11 DIV. Every 3 days, the medium was changed with supplemented Neurobasal medium without glutamate.

#### 4.4.2 HT22 cell culture

Mouse hippocampal‐derived HT22 cell line, a neuronal cell line lacking NMDAR (Maher & Davis, 1996), was generously acquired from Dr. Dave Schubert (Salk Institute). Cells were cultured in high‐glucose DMEM D5648 supplemented with 10% heat‐inactivated FBS, 12 mM NaHCO_3_, 5 mM Hepes and 100 μg/mL penicillin–streptomycin, pH 7.3 in T‐75 culture flasks in a humidified incubator with 5% CO_2_ and 95% air, at 37°C. Sub‐cultures were obtained after a 2 min dissociation step using a Ca^2+^/Mg^2+^‐free dissociation medium containing 140 mM NaCl, 1.47 mM KCl, 8.1 mM Na_2_HPO_4_, 1.47 mM KH_2_PO_4_, 0.55 mM EDTA, pH 7.3 at 37°C. Cells were then centrifuged at 800 rpm for 5 min and the pellet resuspended in fresh culture medium. Cells were cultured at a density of 9 × 10^3^ cells/cm^2^ for 48 h or 3 × 10^3^ cells/cm^2^ for 72 h in 6, 12, or 96‐well plates or 18 mm coverslips (in 12‐well plates).

### 4.5 Cell treatment

Hippocampal neurons at 10–13 DIV were treated for 24 h with HDACis, namely SB, SAHA, and Tac. WT neurons were also treated for 23 h with 1 μM AβO following 1 h pre‐incubation with Tac. On the day after plating, HT22 cells were pre‐exposed for 1 h with HDACis and then co‐treated with 1 μM AβO for the remaining 23 h of incubation. To induce selective HDACs knockdown, siRNA‐calcium phosphate precipitates containing 80 pmol of the indicated siRNA duplex were added to each well containing culture medium on the day following plating. Scrambled siRNA was used as a control. After 5 h incubation, culture medium was changed to fresh medium. On the third day after plating, cells were treated with 1 μM AβO and incubated for additional 24 h. For NFAT transcriptional activity studies, hippocampal neurons or HT22 cells were transfected with 0.75 μg of NFAT luciferase reporter for 48 h prior to analyses and treated with Tac plus AβO for 24 h.

### 4.6 Human samples

Human temporal cortex was obtained from the Neurological Tissue Bank, Biobanc Hospital Clinic‐IDIBAPS, as previously described (Esteves & Cardoso, 2020), from AD patients at Braak stages III–IV (*n* = 5; age range 74–89) and age‐matched cognitively unimpaired controls (*n* = 6; age range 63–76). The study was approved by the local regional ethical committee (Law 14/2007 on Biomedical Research).

Peripheral blood mononuclear cells (PBMCs) from 26 participants were obtained as part of a previous study (Mota et al., 2015). Briefly, patients in the clinical continuum of AD and healthy age‐matched controls were selected from the ageing‐cohort of the Memory Clinic (Neurology Unit) of the Centro Hospitalar e Universitário de Coimbra (CHUC) where they are routinely and prospectively assessed with comprehensive neuropsychological, full biomarker characterization and ApoE genotyping. Patients included individuals (*n* = 5, age range 59–76) fulfilling the established criteria for MCI due to AD according to standard criteria (Albert et al., 2011) and patients with dementia due to AD (McKhann et al., 2011) in the various stages of severity: mild AD (*n* = 5, age range 58–83), moderate AD (*n* = 5, age range 64–85), and severe AD (*n* = 4, age range 62–89). Age‐matched cognitively‐healthy volunteers (*n* = 7, age range 55–78) confirmed by formal neuropsychological assessment were also recruited as controls; exclusion criteria for all these participants are history of neurologic or psychiatric diseases, including major depressive disorder in the last 6 months, history of severe/chronic unstable disease or substance abuse or medication that interferes with the biochemistry. Samples were previously obtained from peripheral venous blood and stored in liquid N_2_. The study was approved by the Ethics Committee of CHUC and written informed consent was obtained from all subjects prior to inclusion in the study.

### 4.7 Cell metabolic activity, proliferation, and viability assays

For cell metabolic activity assay, hippocampal neurons and HT22 cells were incubated with 0.5 mg/mL MTT in sodium‐based medium (Na^+^ medium containing: 140 mM NaCl, 5 mM KCl, 1 mM CaCl_2_, 1 mM MgCl_2_, 10 mM glucose, 10 mM Hepes, pH 7.4) or Krebs medium (135 mM NaCl, 5 mM KCl, 1.8 mM CaCl_2_, 0.4 mM KH_2_PO_4_, 1 mM MgSO_4_, 5.5 mM glucose, 20 mM Hepes, pH 7.4), respectively, for 2 h at 37°C. The resulting formazan crystals were dissolved in acidic‐isopropanol (0.04 M HCI in isopropanol). Absorbance was measured at 570 nm using a SpectraMAX Plus microplate reader (Molecular Devices). MTT reduction extent for each condition was normalized to the corresponding control.

To evaluate HT22 cells proliferation, cells were detached from 12‐well plates at each timepoint, resuspended in fresh culture medium and then diluted in 0.4% Trypan blue solution. Viable cells (unstained cells) were counted under an inverted microscope.

For viability assay, cells were subjected to double staining detection method by using Hoechst/PI to distinguish normal, apoptotic and necrotic cells. Cells were incubated with 2 μg/mL Hoechst 33342, a blue‐fluorescence dye (excitation/emission ~350/461 nm when bound to DNA) and 2 μg/mL PI, a red‐fluorescence dye (excitation/emission ~535/617 nm when bound to DNA), in the dark for 3 min at room temperature, then washed with Krebs medium and further examined and scored using the Axioscope 2 Plus upright microscope (Zeiss). Nuclear condensation was used to distinguish apoptotic cells. PI‐positive cells were identified as necrotic cells.

### 4.8 Mitochondrial membrane potential measurement

Hippocampal neurons and HT22 cells were incubated with 100 nM TMRM^+^ dye (quench mode) plus 0.2% (v/v) pluronic acid in Na^+^ medium or Krebs medium, respectively, for 30 min at 37°C. To directly assess mitochondrial function, glycolysis was inhibited by replacing glucose with 2 mM 2‐deoxy‐d‐glucose and by adding 10 mM pyruvate, in order to directly feed mitochondria (“pyruvate‐based medium”). Basal fluorescence (540 nm excitation and 590 nm emission) was measured using Spectrofluorometer Gemini EM (Molecular Devices) or SpectraMax iD3 (Molecular Devices). Maximal mitochondrial depolarization was also performed in every individual experiment by adding oligomycin (2 μg/ml) to prevent ATP synthase reversal and a protonophore (2 μM FCCP). Results were expressed as the difference between the increase in TMRM^+^ fluorescence upon addition of FCCP plus oligomycin and basal fluorescence values. All plotted values were normalized for initial baseline value.

### 4.9 Seahorse analysis

O_2_ consumption rate (OCR) was measured by using the Seahorse XF24 flux analyzer (Agilent) as previously described (e.g., Ferreira et al., 2018). In brief, neurons were rinsed and equilibrated for 1 h at 37°C in DMEM D5030 supplemented with 2 g/L glucose, 0.2 g/L glutamine, and 0.1 g/L pyruvate, pH 7.4. Then, oligomycin (1 μg/mL), FCCP (1 μM), and antimycin A (1 μM) plus rotenone (1 μM) were sequentially added to evaluate basal respiration, maximal respiration induced by FCCP, ATP synthesis, spare respiratory capacity, H^+^ leak, coupling efficiency, and non‐mitochondrial respiration. Results are expressed in pmol O_2_/min.

### 4.10 Analysis of intracellular free Ca^2+^ levels

Hippocampal neurons and HT22 cells were incubated with 5 μM Fura2‐AM or 1 μM Rhod2‐AM plus 0.2% (v/v) pluronic acid in Na^+^ medium or Krebs medium, respectively, for 30–40 min at 37°C. Neuronal experiments were performed in the absence of Mg^2+^ to maximize NMDAR activation in a pyruvate‐based medium containing 20 μM glycine. Following stimulation with 100 μM NMDA, the neurons were submitted to 10 μM MK‐801, a non‐competitive NMDAR antagonist. Maximal mitochondrial depolarization was achieved by the addition of protonophore FCCP (2 μM) in the presence of 2 μg/mL oligomycin to prevent ATP hydrolysis. In HT22 cells, mitochondrial Ca^2+^ content was also evaluated under maximal mitochondrial depolarization. Calibration of intracellular free Ca^2+^ (Ca^2+^_i_) responses was performed at the end of every individual experiment upon addition of 2 μM ionomycin. ER Ca^2+^ content in both neurons and HT22 cells was estimated following exposure to 1 μM thapsigargin in Ca^2+^‐free medium. Fura2 fluorescence was measured at 340/380 nm excitation and 510 nm emission, and Rhod2 fluorescence at 552 nm excitation and 581 nm emission using Spectrofluorometer Gemini EM (Molecular Devices) or SpectraMax iD3 (Molecular Devices). All plotted values were normalized for initial baseline value.

### 4.11 Animal treatment

APP/PS1 versus WT females aged 9 months old were treated daily with intraperitoneal injections of Tac (30 mg/kg/day; *n* = 7/group) or vehicle (DMSO: PEG: Saline [5:30:65]; *n* = 9/group) for 30 days. The body weight of all mice was measured and recorded once a week.

### 4.12 Brain tissue preparation

On 30th day approximately 30–40 min after Tac/vehicle administration, mice were anesthetized intraperitoneally with 80 mg/kg pentobarbital and then perfused transcardially with 20 mL saline solution (0.9% NaCl). For protein/RNA samples, hippocampi were quickly dissected, frozen in liquid nitrogen, and stored at −80°C until further extraction. For the immunohistochemical analysis, the hemisphere was removed, fixed with 4% paraformaldehyde at 4°C for 24 h, cryoprotected in 30% sucrose at 4°C until it sank, frozen in liquid nitrogen and stored at −80°C until use. Brain blocks embedded in OCT medium were sectioned coronally on a cryostat at a thickness of 40 μm and collected onto SuperFrost adhesion slides.

### 4.13 Protein extraction and Western blotting

Human PBMCs, hippocampal neurons, and HT22 cells were lysed in ice cold RIPA buffer (150 mM NaCl, 50 mM Tris–HCl, 5 mM EGTA, 1% Triton X‐100, 0.5% sodium deoxycholate, 0.1% SDS) supplemented with 1 mM dithiothreitol, 1 mM PMSF, and 1 μg/mL protease inhibitor cocktail containing chymostatin, pepstatin A, leupeptin, and antipain. Following a freeze–thaw cycle, cellular extracts were centrifuged at 14,000 rpm using an Eppendorf Centrifuge 5417R for 10 min at 4°C and protein quantification performed by the BioRad protein assay. Human cortex and mouse hippocampi were homogenized in pre‐boiled 1% SDS and boiled at 100°C for 10 min. After centrifugation, protein content was quantified using BCA protein assay. Equivalent amounts of protein samples (15–30 μg) were denatured with sample buffer (containing 50 mM Tris–HCl pH 6.8, 2% SDS, 5% glycerol, 100 mM dithiothreitol, 0.01% bromophenol blue) at 95°C for 5 min and separated on a 7.5%–15% SDS‐PAGE. Proteins were then transferred onto PVDF membranes and blocked with 5% BSA or non‐fat dry milk in Tris‐buffered saline (25 mM Tris–HCl, 150 mM NaCl, pH 7.6) plus 0.1% Tween‐20 (TBS‐T) according to the manufacturer's recommendation for 1 h at room temperature. Membranes were incubated with primary antibodies against acetyl‐H3 (0.1 μg/mL; 17 kDa), H3 (1:1000; 17 kDa), InsP3R1 (1:300; 290 kDa), GRP75 (1:1000; 75 kDa), VDAC1 (1 μg/mL; 39 kDa), HDAC2 (1:1000; 60 kDa), and HDAC3 (1:1000; 49 kDa) overnight at 4°C or β‐actin (1:20,000; 42 kDa) and β‐tubulin (1:20,000; 55 kDa) for 1 h at room temperature. After a washing step with TBS‐T, membranes were incubated with the respective secondary antibodies (1:10,000) for 1 h at room temperature. Membranes were washed in TBS‐T, further incubated with ECF substrate and immunoreactive bands visualized on a BioRad ChemiDoc Touch Imaging System and further quantified using Image Lab analysis software (BioRad).

### 4.14 RNA isolation and quantitative real time PCR (qRT‐PCR)

Total RNA was extracted from HT22 cells or mouse hippocampi using NZYol (1 mL per 10 cm^2^ growth area or 10 mg tissue), according to the instructions of the supplier. Briefly, lysates were incubated for 5–10 min at room temperature and chloroform (200 μL per 1 mL NZYol used) was added and mixed vigorously before incubating for another 3 min. After centrifugation at 12,000 *g* for 15 min at 4°C, the clear upper aqueous layer was transferred to a new tube, isopropanol (500 μL per 1 mL NZYol) was added and the samples incubated for 10 min at −20°C before centrifugation at 12,000 *g* for 10 min, at 4°C. The RNA precipitate was washed with 75% ethanol and the samples were centrifugated at 12,000 *g* for 10 min, at 4°C. The pellet was dried at room temperature for 10 min and then solubilized in DEPC‐treated water. RNA concentration was determined with NanoDrop 2000c spectrophotometer (Thermo Scientific). cDNA was synthesized from 500 ng of total extracted RNA using the NZY First‐Strand cDNA Synthesis Kit, following the manufacturer instructions. PCR reactions were performed in 10 μL volumes containing 5 μL of iQ SYBR Green Supermix, 300 nM of each primer and 50 ng of cDNA template in a BioRad CFX96 Real‐Time PCR Detection System using the following cycling conditions: initial denaturation at 95°C for 3 min, followed by 40 cycles of denaturation at 95°C for 15 s and annealing at 60°C for 45 s. At the end, samples were subjected to a melting curve analysis in order to confirm the absence of unspecific amplification products and primers dimers. Samples containing no template were included as negative controls in all experiments. Reactions were run in duplicates. Analysis of gene expression was performed using the ΔΔCT method. Actin was used as an internal control for all samples. PCR primer sequences used were as follows (forward primer/reverse primer):

*Insp3r*: 5′‐GTATGCGGAGGGATCTACGA/5′‐AACACAACGGTCATCAACCA
*Sigmar1*: 5′‐ACCAATGGAAAGAGGGCAC/5′‐AACAGGGTAGACGGAATAACAC
*Grp75*: 5′‐CAGGCAGCATCTTCCCTACAG/5‐CCAGTGCCAGAACTTCCAGAA
*Vdac1*: 5′‐AAGTGAACAACTCCAGCCTGA/5′‐CACCAGCATTGACGTTCTTG
*Mcu*: 5′‐CGCCAGGAATATGTTTATCCA/5′‐CTTGTAATGGGTCTCTCAGTCTCTT
*Pdzd8*: 5′‐TCAACTGATGGGTATGCTGG/5′‐ATAGCAATGAGCCGATCTCC
*Vapb*: 5′‐GAAGGTGATGGAAGAGTGCAG/5′‐CCCGAAGTCCGTCTTCTTC
*Rmdn3:* 5′‐GGTCCGCTCTCATATAGAAGAGAAC/5′‐CCTCTCTCTGGCAAAAGGGAAC
*Mfn2:* 5′‐TCCTCTGTTCCAGTTGTA/5′‐TCGCTTATCCTTCTTGAC
*Mfn1:* 5′‐GCAGACAGCACATGGAGAGA/5′‐GATCCGATTCCGAGCTTCCG
*Actin*: 5′‐GTGACGTTGACATCCGTAAAGA/5′‐GCCGGACTCATCGTACTCC

### 4.15 Proximity ligation assay (PLA)

Cells were fixed with 4% paraformaldehyde at room temperature for 15 min and permeabilized with 0.5% Triton X‐100 for 5 min and in situ Duolink PLA was then performed using 40 μL of solution per 1 cm^2^ sample on a slide according to manufacturer's instruction. In brief, the samples were incubated with blocking buffer at 37°C for 1 h in a humidified chamber, followed by incubation with primary antibodies against InsP3R1 (1:50) and VDAC1 (1:50) overnight at 4°C. The slides were rinsed in wash buffer, the oligo‐linked secondary probes (1:5) anti‐mouse MINUS and anti‐rabbit PLUS were added and the samples were incubated at 37°C for 1 h in a humidified chamber. After a washing step, samples were incubated with ligase (1:40) for 30 min at 37°C, further rinsed in wash buffer and then incubated with polymerase (1:80) in amplification buffer for 100 min at 37°C. Finally, the slides were mounted in mounting media containing DAPI. Fluorescence signal amplification was recorded as a Z‐stack using the Zeiss LSM 710 confocal laser scanning microscopy under a 63x/1.4 objective and analyzed using the IMARIS analysis software.

### 4.16 Transmission electron microscopy (TEM)

HT22 cells were collected and centrifuged at 2500 rpm for 5 min and the pellets fixed in 2.5% (v/v) glutaraldehyde in 0.1 M phosphate buffer (pH 7.4) for 2 h at room temperature. Following rinsing in the same buffer, postfixation was performed using 1% (w/v) osmium tetroxide for 1 h. After rinsing in phosphate buffer and distilled water, 1% (w/v) aqueous uranyl acetate was added to the cells during 1 h for contrast enhancement. Following embedding in 2% (w/v) molten agar, samples were dehydrated in a graded ethanol series (30%–100%), impregnated and embedded in Epoxy resin. Ultrathin sections (70 nm) were mounted on copper grids and stained with 0.2% (w/v) lead citrate for 7 min. Observations of 10 independent cells per condition were carried out on a FEI Tecnai G2 Spirit BioTwin electron microscope at 100 kV. Mitochondria‐ER contacts sites (MERCS) were considered when the distance between ER and mitochondria was less than 25 nm. MERCS length, MERCS distance, number of MERCS per mitochondria, mitochondrial area, perimeter, Feret's diameter (defined as longest distance between any two points in the mitochondria), and aspect ratio (defined as the ratio between major and minor mitochondrial axis) were quantified using image analysis software ImageJ (Fiji) according to Lam et al., 2021.

### 4.17 Immunohistochemistry

Tissue sections were heated in sodium citrate buffer (10 mM sodium citrate, 0.05% Tween‐20, pH 6.0) for 10 min for antigen retrieval. After cooling, the slides were rinsed with PBS and blocked with 0.3% Triton X‐100/3% BSA for 1 h at room temperature. Sections were incubated with primary antibodies against Aβ_1–42_ (1:200) and APP (1:500) overnight at 4°C, then washed with PBS and incubated with the respective secondary antibodies (1:1000) for 1 h at room temperature. Finally, nuclei were counterstained with 1 μg/mL Hoechst 33342 for 5 min. Fluorescent photomicrographs of whole cross‐sectional hippocampal regions were serial scanned under a 20× objective using a Zeiss Axio Scan Z1 microscope slide scanner and fluorescence levels quantified by using image analysis software ImageJ (Fiji).

### 4.18 Luciferase reporter assay

Cells were collected in ice‐cold lysis buffer (1.15 M Tris, 1 mM EDTA, 8 mM MgCl_2_, 15% glycerol, 1 mM DTT, 1% Triton X‐100, pH 7.4) after a freeze–thaw cycle and then samples were transferred to a 96‐well white opaque plaque. Luminescence was measured with 10 s integration time after addition of reading buffer (1.15 M Tris, 1 mM EDTA, 8 mM MgCl_2_, 15% glycerol, 1 mM DTT, 2 mM ATP‐Mg^2+^, pH 7.4) and luciferin (50 mg/mL; pH 8) in a LMAX II 384 Luminometer (Molecular Devices). Data were normalized to protein content.

### 4.19 Statistical analysis

Data were analyzed by GraphPad Prism 8 software and expressed as the mean of at least three independent experiments ± SEM. Statistical significance was assessed using the Mann–Whitney test, unpaired Student's t‐test, Kruskal–Wallis followed by uncorrected Dunn's multiple comparison test or one‐way ANOVA followed by uncorrected Fisher's LSD multiple comparison test. Sample normality was tested using Shapiro–Wilk test. Statistical significance was defined as *p < 0.05, **p < 0.01, ***p < 0.001, and ****p < 0.0001.

## AUTHOR CONTRIBUTIONS

DM acquired and analyzed the data. ILF and ACR conceptualized and designed the study. DM, ILF and ACR interpreted the results. DM wrote the paper with critical edits from ILF and ACR. RL carried out mice treatment and sacrifice. SMC provided the postmortem human brain tissue. IS contributed in participant's examination and recruitment for blood donation. All authors read, edited, and approved the final version of the manuscript.

## CONFLICT OF INTEREST STATEMENT

The authors confirm that there are no financial or non‐financial competing interests to report.

## CONSENT FOR PUBLICATION

All authors read the manuscript and consent with its publication.

## Supporting information


Figure S1

Figure S2

Figure S3

Figure S4

Figure S5

Appendix S1

## Data Availability

The data that support the findings of this study are available from the corresponding author upon reasonable request.
