# Supplementary figures and images for: Reduction of class I histone deacetylases ameliorates ER‐mitochondria cross‐talk in Alzheimer's disease
Source: Aging Cell. 2023 Jun 26;22(8):e13895. doi: 10.1111/acel.13895 (PMC10410063; doi:10.1111/acel.13895)

Figure S1

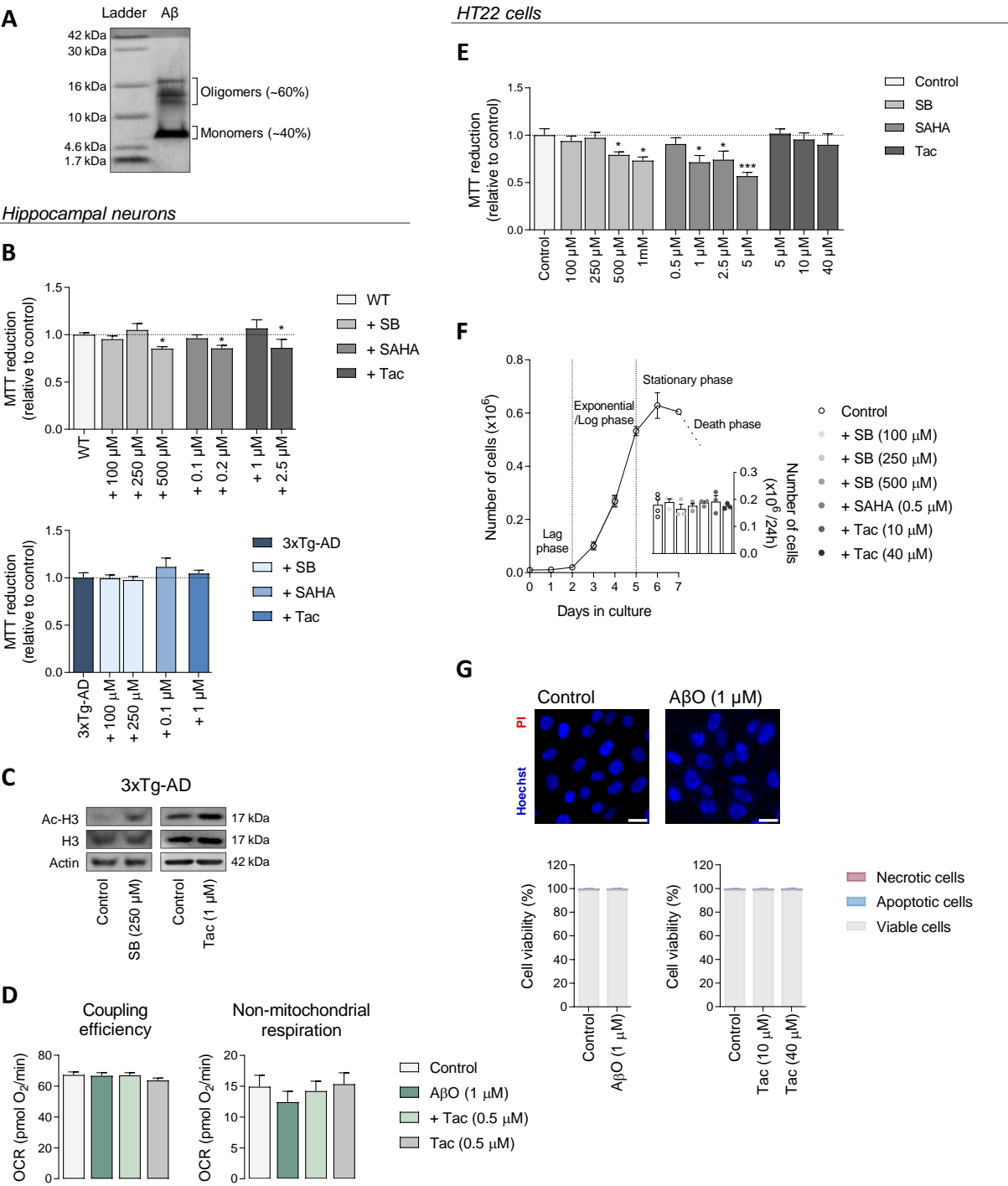

Supplement: Supplementary file 1 — Figure S1 [file ACEL-22-e13895-s005.pdf]

Hippocampal neurons

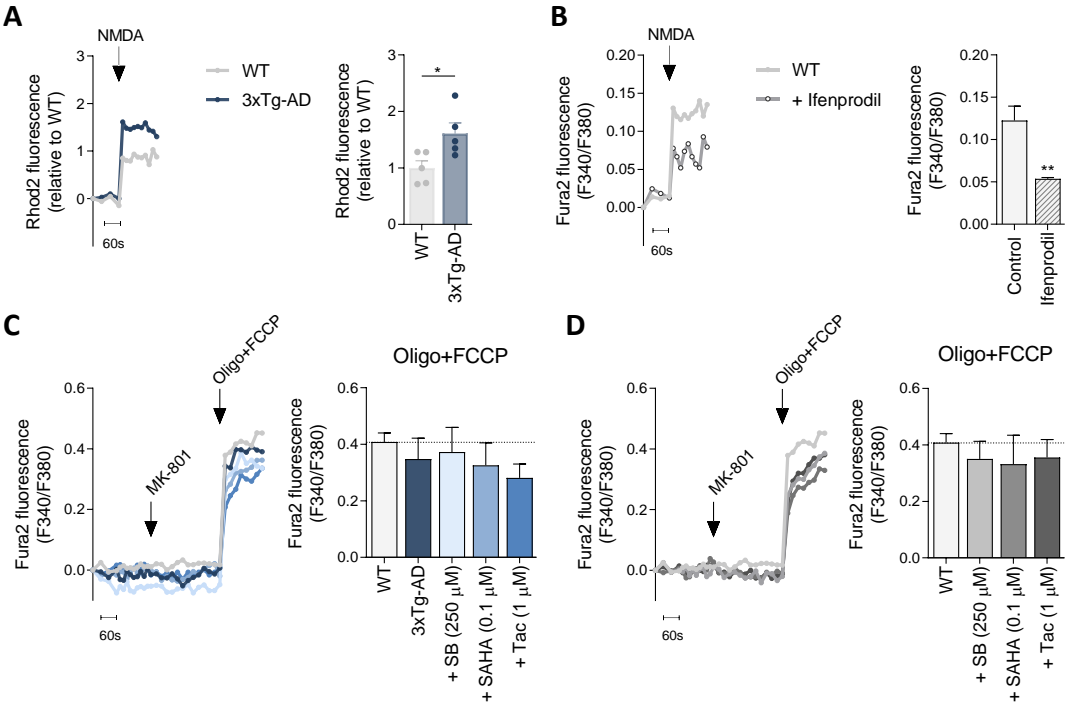

HT22 cells

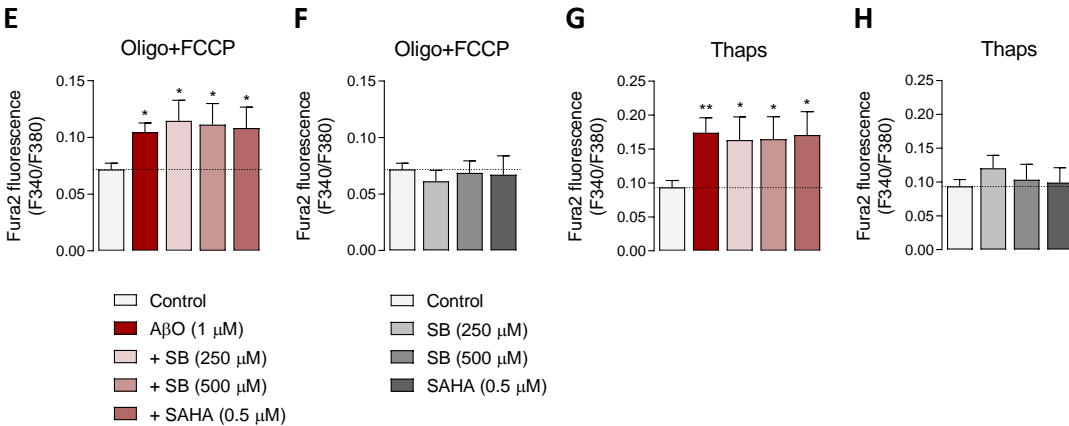

Supplement: Supplementary file 2 — Figure S2 [file ACEL-22-e13895-s004.pdf]

Figure S3

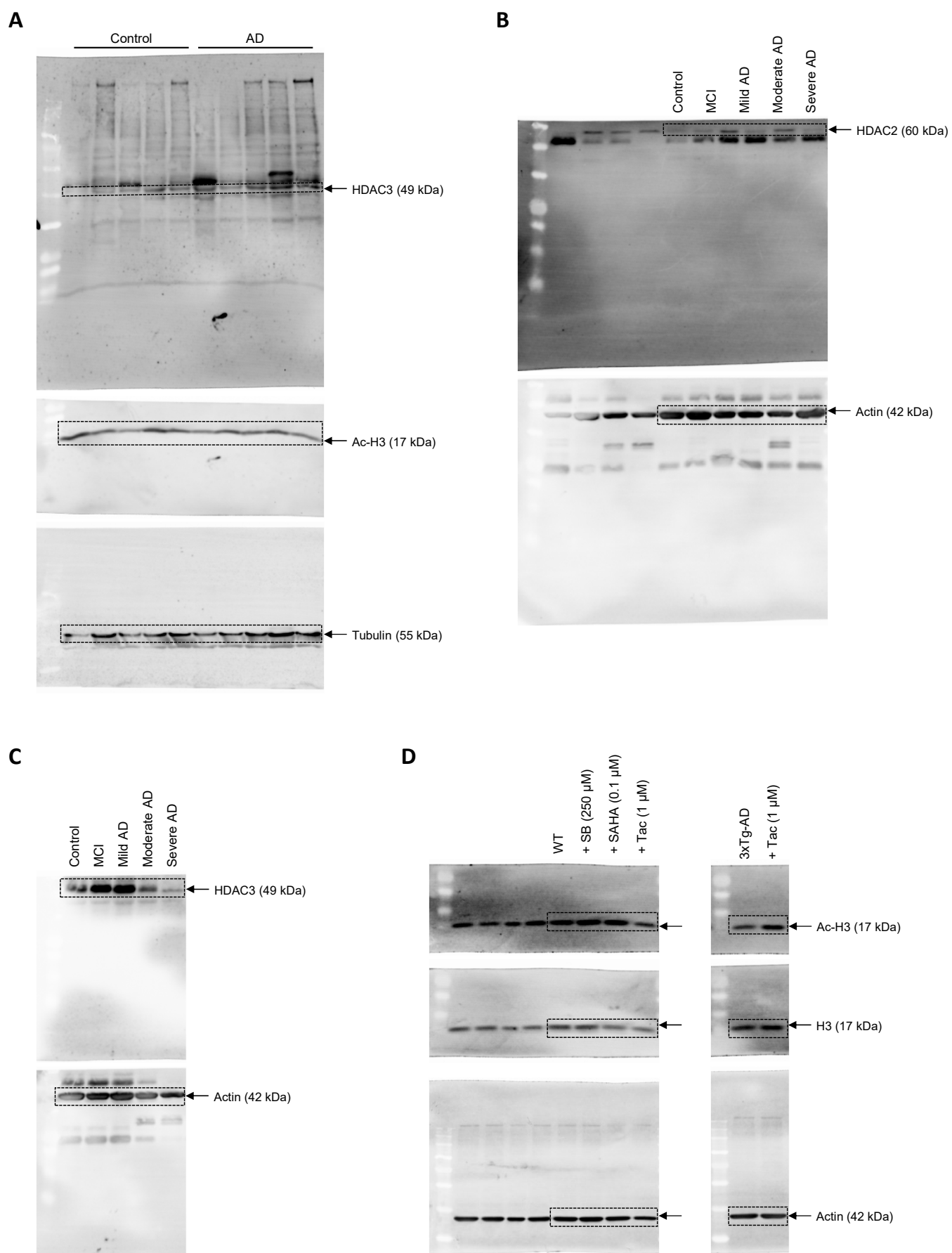

Supplement: Supplementary file 3 — Figure S3 [file ACEL-22-e13895-s002.pdf]

Figure S4

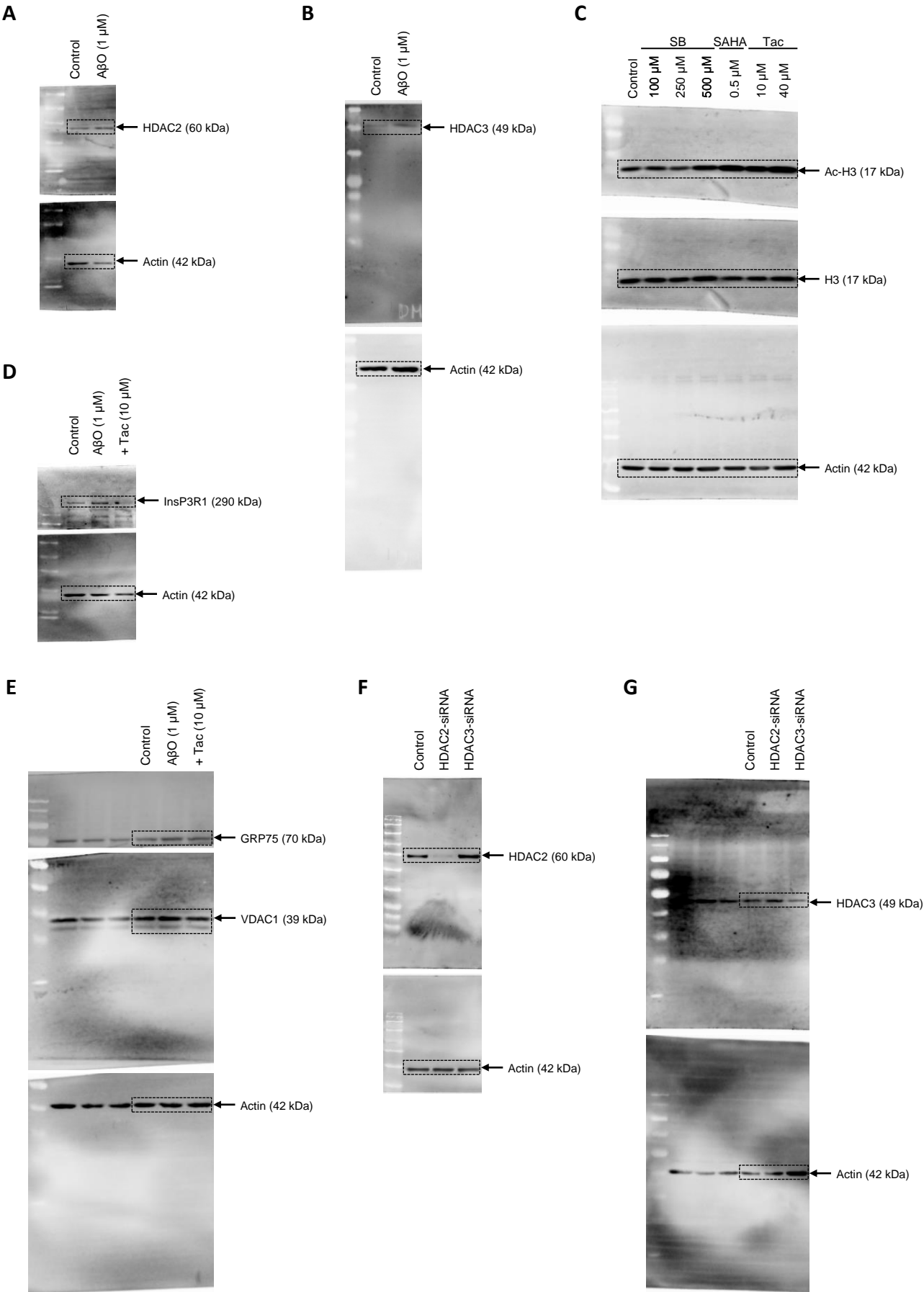

Supplement: Supplementary file 4 — Figure S4 [file ACEL-22-e13895-s003.pdf]

Figure S5

**A**

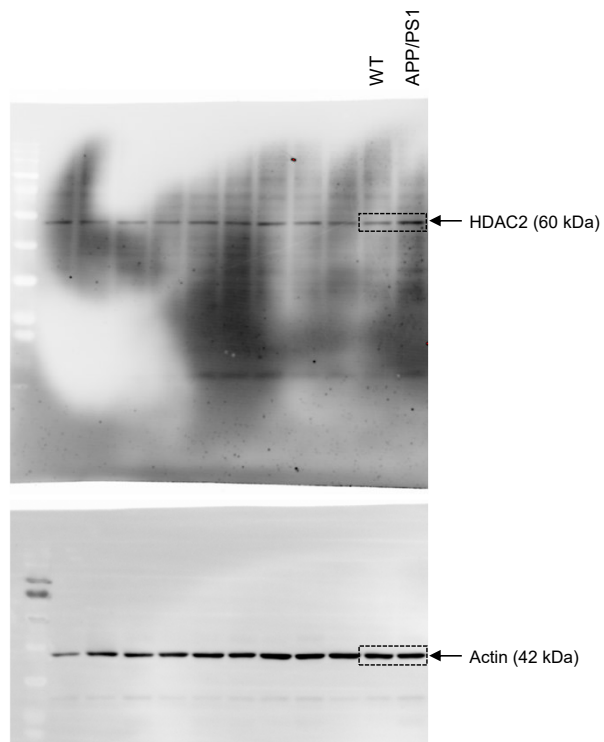

**B**

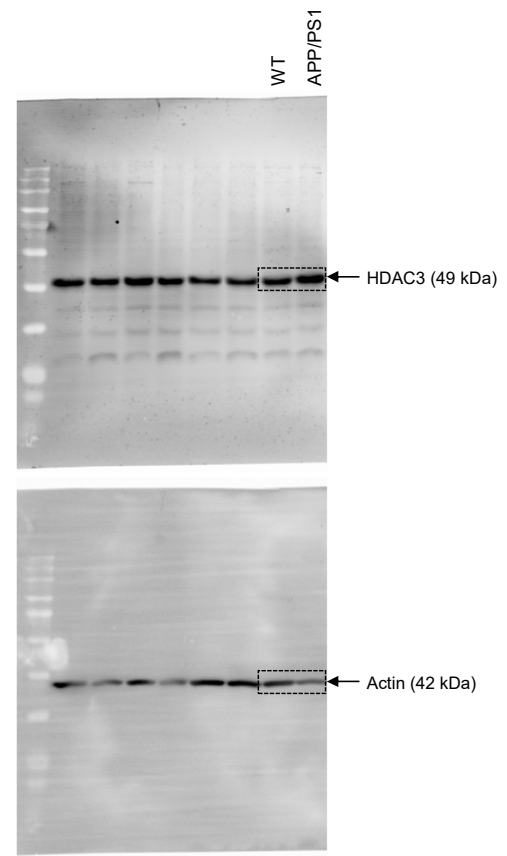

**C**

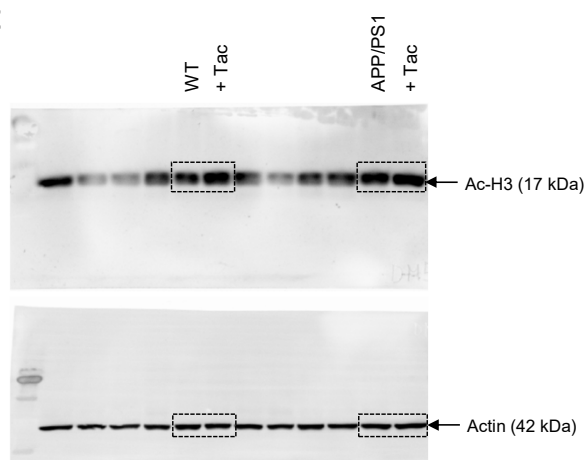

Supplement: Supplementary file 5 — Figure S5 [file ACEL-22-e13895-s001.pdf]
