# Supplementary material for: Reduction of class I histone deacetylases ameliorates ER‐mitochondria cross‐talk in Alzheimer's disease
Source: Aging Cell. 2023 Jun 26;22(8):e13895. doi: 10.1111/acel.13895 (PMC10410063; doi:10.1111/acel.13895)
Supplement: Supplementary file 6 — Appendix S1 [file ACEL-22-e13895-s006.docx]

**Fig. S1** Screening of HDACis concentrations in hippocampal neurons and HT22 cells. Representative gel of Aβ_1-42_ preparation obtained from synthetic Aβ_1–42_ as described in Materials and methods (**A**). Hippocampal neurons were treated for 24 h with HDACis (250 μM SB; 0.5 μM SAHA or 1 μM Tac). Cell metabolic activity was measured using the MTT assay (**B**). Western blotting analysis of acetyl-H3 protein levels in 3xTg-AD hippocampal neurons following 24 h incubation with SB (250 µM) or Tac (1 µM) (**C**). WT hippocampal neurons were pre-treated for 1 h with Tac (0.5 µM) and then co-incubated with 1 μM AβO for the remaining 23 h. Coupling efficiency and non-mitochondrial respiration were quantified using a Seahorse analyzer (**D**). HT22 cells were treated for 24 h with HDACis (SB, SAHA and Tac at the indicated concentrations). Cell metabolic activity was measured using the MTT assay (**E**). Cell proliferation was evaluated by cell counting using Trypan blue exclusion method (**F**). Representative images of double Hoechst/PI staining under fluorescence microscopy (scale bar = 20 µm) and quantification of cell viability (180-330 cells per field and 5-6 fields per condition were counted) (**G**). Data are the mean ± SEM of 3-9 independent experiments, run in triplicates to quadruplicates. Statistical analysis: One-way ANOVA followed by uncorrected Fisher’s LSD multiple comparison test; *p<0.05; ***p<0.001 when compared to the control.

**Fig. S2** Effect of HDACis on Ca^2+^_i_ levels in hippocampal neurons and AβO-treated HT22 cells. Hippocampal neurons were stimulated with NMDA (100 µM) in Mg^2+^-free pyruvate-based medium containing glycine (20 µM). Representative Rhod2 fluorescence trace and peak amplitude after NMDA stimulus (**A**). Representative F340/F380 trace and peak amplitude in response to NMDA following a 5 min pre-incubation with ifenprodil (10 µM) (**B**). WT and 3xTg-AD hippocampal neurons were treated for 24 h with HDACis (250 µM SB; 0.1 µM SAHA or 1 µM Tac). Representative F340/380 fluorescence trace and peak amplitude in response to maximal mitochondrial depolarization induced by oligo plus FCCP (2 µg/ml; 2 µM) in 3xTg-AD (**C**) and WT (**D**) neurons. HT22 cells were pre-treated for 1 h with SB (250 or 500 µM) and SAHA (0.5 µM) and then co-incubated with 1 μM AβO for the remaining 23 h of incubation. Representative F340/380 fluorescence trace and peak amplitude after oligo+FCCP stimulation in the presence (**E**) or absence (**F**) of AβO. Representative F340/380 fluorescence trace and peak amplitude in response to ER-Ca^2+^ storage depletion induced by thapsigargin (1 µM) in the presence (**G**) or absence (**H**) of AβO. Data are the mean ± SEM of 3-7 independent experiments, run in triplicates to quadruplicates. Statistical analysis: Kruskal-Wallis followed by uncorrected Dunn’s multiple comparison test, One-way ANOVA followed by uncorrected Fisher’s LSD multiple comparison test, Mann-Whitney test and unpaired Student’s t-test; *p<0.05; **p<0.01 when compared to control.

**Fig. S3** Full-length Western blots for human samples and hippocampal neurons. Full-length blot image for figure 1A,B (**A**), figure 1D (**B, C**) and figure 1J (**D**).

**Fig. S4** Full-length Western blots for HT22 cells. Full-length blot image for figure 1E (**A,B**), figure 1F (**C**), figure 3L (**D**), figure 3M,N (**E**) and figure 5B (**F,G**).

**Fig. S5** Full-length Western blot images for APP/PS1 mice. Full-length blot image for figure 6A (**A,B**) and figure 6C (**C**).
